# The First Pituitary Proteome Landscape From Matched Anterior and Posterior Lobes for a Better Understanding of the Pituitary Gland

**DOI:** 10.1016/j.mcpro.2022.100478

**Published:** 2022-12-05

**Authors:** Arghya Banerjee, Deepatarup Biswas, Abhilash Barpanda, Ankit Halder, Shamira Sibal, Rohit Kattimani, Abhidha Shah, Anita Mahadevan, Atul Goel, Sanjeeva Srivastava

**Affiliations:** 1 Department of Biosciences and Bioengineering, Indian Institute of Technology Bombay, Mumbai, India; 2 Lokmanya Tilak Municipal Medical College, Mumbai, India; 3 Anthony Claret School, Jalahalli, Bengaluru, India; 4 Department of Neurosurgery at King Edward Memorial Hospital and Seth G. S. Medical College, Mumbai, India; 5 Human Brain Bank, National Institute of Mental Health and Neuro Sciences (NIMHANS), Bangalore, India

**Keywords:** LFQ, PRM, uPE1, string and pathway analysis, ACTH, adrenocorticotropic hormone, AVP, arginine vasopressin, CLAT, clathrin, DEP, differentially expressed protein, FA, formic acid, FC, fold change, FDR, false discovery rate, FSH, follicle-stimulating hormone, GO, Gene Ontology, HPP, Human Proteome Project, IGSF1, immunoglobulin superfamily member-1, KEGG, Kyoto Encyclopedia of Genes and Genomes, LFQ, label-free quantitation, LH, luteinizing hormone, METTL26, methyltransferase-like 26, MS, mass spectrometry, NPTX2, neuronal pentraxin-2, OXT, oxytocin–neurophysin, PCOLCE2, procollagen C-endopeptidase enhancer-2, POU1F1, pituitary-specific positive transcription factor 1, PD2.4, Proteome Discoverer 2.4, PDB, Protein Data Bank, POMC, pro-opiomelanocortin, PRL, prolactin, PRM, parallel reaction monitoring, PSM, peptide spectral match, TGFBR3L, transforming growth factor-beta receptor type 3-like protein, TSH, thyroid-stimulating hormone

## Abstract

To date, very few mass spectrometry (MS)–based proteomics studies are available on the anterior and posterior lobes of the pituitary. In the past, MS-based investigations have focused exclusively on the whole pituitary gland or anterior pituitary lobe. In this study, for the first time, we performed a deep MS-based analysis of five anterior and five posterior matched lobes to build the first lobe-specific pituitary proteome map, which documented 4090 proteins with isoforms, mostly mapped into chromosomes 1, 2, and 11. About 1446 differentially expressed significant proteins were identified, which were studied for lobe specificity, biological pathway enrichment, protein–protein interaction, regions specific to comparison of human brain and other neuroendocrine glands from Human Protein Atlas to identify pituitary-enriched proteins. Hormones specific to each lobe were also identified and validated with parallel reaction monitoring–based target verification. The study identified and validated hormones, growth hormone and thyroid-stimulating hormone subunit beta, exclusively to the anterior lobe whereas oxytocin–neurophysin 1 and arginine vasopressin to the posterior lobe. The study also identified proteins POU1F1 (pituitary-specific positive transcription factor 1), POMC (pro-opiomelanocortin), PCOLCE2 (procollagen C-endopeptidase enhancer 2), and NPTX2 (neuronal pentraxin-2) as pituitary-enriched proteins and was validated for their lobe specificity using parallel reaction monitoring. In addition, three uPE1 proteins, namely THEM6 (mesenchymal stem cell protein DSCD75), FSD1L (coiled-coil domain–containing protein 10), and METTL26 (methyltransferase-like 26), were identified using the NeXtProt database, and depicted tumor markers S100 proteins having high expression in the posterior lobe. In summary, the study documents the first matched anterior and posterior pituitary proteome map acting as a reference control for a better understanding of functional and nonfunctional pituitary adenomas and extrapolating the aim of the Human Proteome Project towards the investigation of the proteome of life.

## Highlights

• MS-based analysis of both matched anterior and posterior lobes of healthy pituitary.
• Building the first proteome database of the anterior and posterior lobes.
• Studying proteins and hormones specific to each lobe of the pituitary and their validation with PRM.
• Studying pituitary-enriched proteins, their lobe specificity, and their target verification with PRM.

## Graphical Abstract

**Figure:**
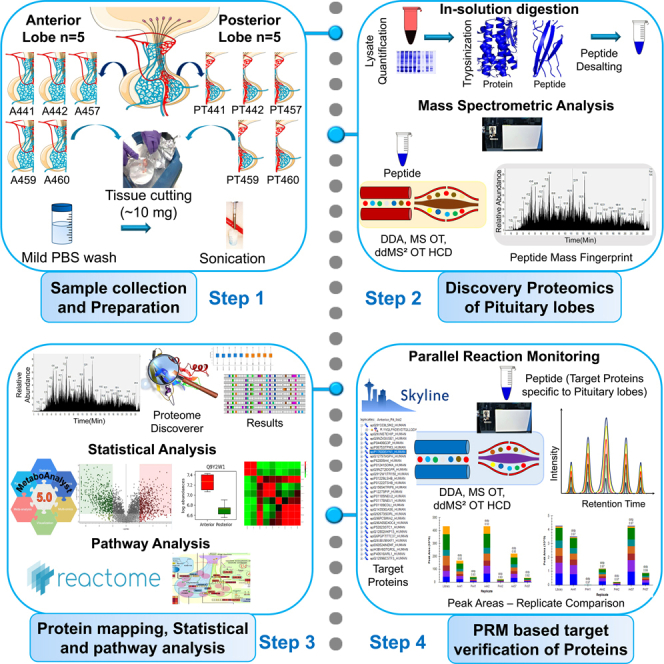

## In Brief

Our study of the first pituitary proteome draft offers the following novelty; for the first time, we present an extensive proteomic database containing proteins from both the anterior and posterior lobes of healthy pituitary, identifying various pituitary-enriched proteins and their lobe specificities, followed by target verification of pituitary hormones; for the first time, we are describing three uPE1 and have demonstrated that S100 proteins, which are known markers for pituitary adenomas, showed their presence significantly in the posterior lobe.

The pituitary is a pea-sized endocrine gland attached to and located below the hypothalamus. It is known as the master endocrine gland because of its various regulatory functions. It consists of histologically distinct anterior glandular tissue mass, adenohypophysis, and posterior neural neurohypophysis. These lobes show different embryological origins (dual origin), having differences in morphological and anatomical features. Adenohypophysis originates from the oral ectoderm, and neurohypophysis develops from the neural ectoderm (1). Further distinct signatures in the genomic and proteomic expression profile make the lobes unique and peculiar regarding functions, regulations, and organizations. The major differences among the lobes include their constitutive cells, hormone synthesis, stimulating factors, connective tissue components, and various other endocrinological roles.

The adenohypophysis consists of five cellular subtypes: somatotropes, lactotropes, corticotropes, thyrotropes, and gonadotropes. Each of these cells is characterized by the hormones they produce and the biological process they regulate. Neurohypophysis is mainly composed of magnocellular axon terminals that synthesize peptide hormones oxytocin and vasopressin. The hormones produced by the pituitary gland act on other endocrine organs *via* hypothalamic–pituitary–end-gland axes and relay multilevel signaling to integrate the neural and endocrinal components. The anterior lobe of the pituitary is mainly involved in the secretion of growth hormone (GH), adrenocorticotropic hormone (ACTH), thyroid-stimulating hormone (TSH), follicle-stimulating hormone (FSH), and luteinizing hormone (LH) from five different endocrine cells (somatotrophs, gonadotrophs, lactotrophs, corticotrophs, and thyrotrophs), whereas the posterior pituitary is involved in the secretion of nanopeptide arginine vasopressin (AVP) and oligopeptide oxytocin–neurophysin 1 (OXT). GH coordinates the aspects of nutrition, reproduction, osmoregulation, immune system function, and most importantly, regulates growth and metabolism (particularly lipid metabolism) (2). TSH stimulates endocrine activity in the thyroid gland, stimulating cellular metabolic processes. Pro-opiomelanocortin (POMC) is the prohormone for ACTH, melanocyte-stimulating hormone, and β-endotropin, with multiple regulatory roles in carbohydrate, protein, and lipid metabolism. Prolactin (PRL), known for stimulating lactation, has additional functions related to behavior and homeostasis (3). AVP shows antidiuretic action by modulating aquaporin expression in nephrons and plays a role in homeostasis, immunomodulation, cognitive function, pain perception, and maintaining blood glucose levels (4), whereas OXT plays a role in uterine contraction, milk secretion, emotional regulation, and natriuretic action (5).

Both lobes are regulated and function differently in various normal and diseased conditions. The regulation and functions of these lobes are still not well understood, and there is an urge to explore the lobes at the systems biology level. To date, studies involving anterior and posterior lobe comparison primarily focus on the anatomical (6) or imaging (7) differences between them or their response to simple serum function tests (8). On the other hand, all previous mass spectrometry (MS)–based studies have been performed on the human pituitary, which has generally focused on profiling the pituitary gland as a whole (9), analysis of the anterior pituitary alone (10), and studying serum sample from disease patients (11, 12). The establishment and leadership of the Human Proteome Project (HPP) have helped complete and establish large-scale databases like NeXtProt (https://www.nextprot.org/tools/peptide-uniqueness-checker), HPA, BrainProt, and Peptide Atlas, which serve as a reference map and foundation platform for researchers around the globe. The addition of a human pituitary proteome map will add a new facet of information and open opportunities for understanding pituitary biology better.

This study aims to provide an MS-based deep proteomic analysis of both anterior and posterior lobes, using paired samples of each individual, to deepen the scientific understanding of their physiochemical differences at a more fundamental level. In addition, the study has identified pituitary markers and lobe-specific enriched proteins of different hormones collating HPA and other repository datasets. Furthermore, the *in silico* analysis has appended lobe-specific pathway enrichment, uPE1 protein identification, and functional clustering, making the first landscape of pituitary proteome map.

## Experimental Procedures

### Experimental Design and Statistical Rationale

The study has been approved by the Institute Ethics Committee, IIT Bombay (IITB-IEC/2019/002). Normal pituitary (anterior and posterior lobes) samples were collected from Human Brain Tissue Repository (National Research Facility), NIMHANS, Bangalore, India, with written informed consent from their next-of-kin and human studies reported in article abide by the Declaration of Helsinki principles.

The pituitary was removed during postmortem using the standard procedure by opening the sella turcica and breaking the posterior clinoids, followed by cutting through the diaphragm sella. The pituitary was sliced into two halves in the horizontal plane in the fresh state. In the horizontal plane, the posterior pituitary is clearly distinguishable from the anterior pituitary by its pale gray and firm texture compared with the reddish-brown staining vascular anterior pituitary. Using a sharp scalpel, the two are separated and frozen separately at −86 °C. The corresponding half was fixed in 10% neutral-buffered formalin and processed for paraffin embedding. Serial 3 to 4 micron thick sections from tissues are stained with hematoxylin & eosin for microscopic examination and immunohistochemistry if required. The frozen portions are provided for proteomic studies.

Five matched pairs of dissected anterior and posterior lobe samples of the pituitary glands provided by the brain bank have been used for the study; these tissues were harvested between 9 and 18 h after death. The current study is intended to identify differential expression levels of genes at the proteome level between two different lobes of the pituitary gland. The study has also followed all the basic principles of randomization and appropriate normalization for technical variances. The candidates that are taken forward are made sure that they are detected in the majority of the samples and are governed by the studies of Cairns *et al.* (13).

### Sample Preparation for Label-Free Quantification–Based MS Analysis

Tissue samples were lysed in urea buffer (8 M urea, 50 mM Tris [pH 8.0], 75 mM NaCl, and 1 mM MgCl_2_). These tissue lysates were quantified by bicinchoninic acid protein quantification. Around ∼30 μg of protein lysate was taken reduced with 20 mM (final concentration) Tris(2-carboxyethyl)phosphine and further incubated at 37 °C for 1 h. After reduction, alkylation was performed using 37.5 mM (final concentration) of iodoacetamide and incubated at 37 °C for 20 min at dark. Trypsinization was done using Pierce trypsin in 1:30 ratio with pH 8 and incubated at 37 °C in a shaking dry bath for 16 h. The sample was quenched after trypsinization with a vacuum centrifuge. The dried samples were then desalted using in-house C18 filter stage tips (14); furthermore, these peptides were dried and stored at −80 °C for further use.

### Peptide Quantification and MS Analysis (label-free quantitation)

Desalted peptides were further quantified using Multiskan GO (Thermo Fisher Scientific) after reconstituting it in 1% (v/v) formic acid (FA) by the Scopes method. Around 1 μg of the peptide was injected in nLC (Thermo nano-LC 1200 system) having precolumn (Thermo Fisher Scientific; P/N 164564, S/N 10694527) and analytical column (Thermo Fisher Scientific; P/N ES803A, S/N 10918620) with LC gradient of 240 min having solvent A (0.1% FA in water) and solvent B (80% acetonitrile in 0.1% FA) water, under a flow rate of 5 μl/min. The eluted peptides were analyzed on Orbitrap Fusion Tribrid Mass Spectrometer (Thermo Fisher Scientific) in data-dependent acquisition, MS OT mode having a resolution of 60,000, the scan range of 375 to 1700 *m/z*, maximum injection time of 50 ms, mass tolerance of 10 ppm, dynamic exclusion duration of 40 s, and ddMS^2^ OT higher energy collisional dissociation having a resolution of 15,000, isolation window of 1.2, with a maximum injection time of 30 ms (14) (supplemental Table S1).

### Label-Free Quantitation Data Analysis with Proteome Discoverer 2.4

Proteins from peptide mass fingerprint data of anterior and posterior lobes of the pituitary gland, generated from Mass Spectrometer, were identified using three search engines, Sequest HT, MSPep Search, and MS Amanda 2.0 integrated into Proteome Discoverer 2.4 (PD2.4) (15) using Human Swiss-Prot database (version: 2020_04), which includes a total of 20,353 proteins. The enzyme used for digestion was kept as trypsin with complete digestion and maximum cleavage site of two; minimum and maximum peptide length was considered as default 6 and 144 amino acids. Precursor and fragment mass tolerance were kept as 10 ppm and 0.05 Da. Carbamidomethylation of cysteine (+57.021464 Da) was set as static modification, whereas oxidation of methionine (+15.994915 Da), phosphorylation at S, T, Y (+79.996 Da), and methionine loss and acetylation (N-terminal, +89.030 Da) were set as dynamic modifications. Target false discovery rate (FDR) for peptide spectral matches (PSMs) and peptides was set at 0.01, and the type of identified peptides was set to “unique.” The proteins were further filtered on the criteria of master proteins, high protein FDR confidence, unique peptides greater than 1, and an integrated contaminant database as false.

### Statistical Data Analysis and Biological Interpretation

The sample-to-sample correlation of normalized intensities from Proteome Discoverer was checked, and the correlation coefficient was used to detect the outlier. The evidence-level information and chromosome mapping data were further collated from NeXtProt (release: 2021_04) using the list of identified proteins. The identified unique peptides were examined using the uniqueness checker of NeXtProt. Abundance (normalized) values from PD2.4 result file were used for paired univariate analysis using MetaboAnalyst 5.0 (Wishart Research Group) (16). The normalized abundances were transformed into log (base 10), after which fold change (FC) and paired two-sample *t* tests were performed. The data analysis was primarily performed in Python, Microsoft Excel, and web applications like Metaboanalyst. The identification of statistically significant proteins was determined using paired Welch's *t* test with a threshold of ≤0.05. The log2FC cutoff was kept at ±1.5 to distinguish the differentially expressed proteins (DEPs) between the anterior and posterior groups. Box plots were plotted with the log2-transformed data, which was calculated on the basis of independent-samples *t* test with Bonferroni correction. Different visualization plots, which include heatmap, volcano plots, and correlation plots, were drawn using Hierarchical Clustering Explorer (version 3.5, Shneiderman & Seo) and Metaboanalyst (version 5.0). The list of significant DEPs was further curated from Brain Atlas of Human Protein Atlas (version 20.1, Science for Life Laboratory) and literature to identify the pituitary-enriched proteins based on consensus normalized expression comparison and proteins of the hormone signaling pathway, respectively. The biological and functional analysis between the groups was performed with the significant DEPs where Reactome (version 78; https://reactome.org/), Kyoto Encyclopedia of Genes and Genomes (KEGG; https://www.genome.jp/kegg/pathway.html), text mining, and literature-based curation were primarily emphasized.

### Biological and Functional Clustering Analysis

The list of significant DEPs was further curated from Brain Atlas of Human Protein Atlas (version 20.1) and literature to identify the pituitary-enriched proteins based on consensus normalized expression comparison and proteins of the hormone signaling pathway, respectively. Furthermore, Reactome (version 78) and KEGG were used to map the biological pathways. The protein–protein interaction analysis of DEPs was carried out in STRING (version 11.5, Global Biodata Coalition and ELIXIR) with the physical subnetwork as network type and an interaction score of 0.7 (high). Furthermore, the lobe-enriched proteins were further taken for enrichment clustering in DAVID (Laboratory of Human Retrovirology and Immunoinformatics (LHRI)) (17). The similarity and enrichment threshold were kept as default to export the count, FDR, Bonferroni–Šidák *p* value, population total, and population hits against each enriched cluster. Finally, the enriched Gene Ontology (GO) terms were taken to NaviGO tools (18) for visualization and functional similarity.

### Parallel Reaction Monitoring Assay

Parallel reaction monitoring (PRM) of the target proteins was performed using an Orbitrap Fusion mass analyzer (Thermo Fisher Scientific). PRM method development and optimization of target proteins were performed using Skyline (version 21.2.0.536) (19). The PRM assay was run with a method duration of 60 min, with MS OT mode having a resolution of 60,000, scan range 350 to 1700 (*m/z*), and maximum injection time of 50 ms and tMS^2^ OT higher energy collisional dissociation having isolation window of 1.6, resolution of 30,000, scan range of 50 to 2000, and maximum injection time of 40 ms (supplemental Table S2). The isolation list of the selected proteins was generated from Skyline (20) with the Human Swiss-Prot database (version: 2020_04) and library generated using .pdResult from PD2.4. Around 1 μg of the peptide was injected into nLC with a gradient of 60 min, with solvent A comprising 0.1% FA in water and solvent B comprising 80% acetonitrile in 0.1% FA water and a flow rate of 5 μl/min.

### Isolation List Preparation and Data Analysis

Isolation list preparation and method development for PRM based on identified proteins were validated using Skyline. The human-reviewed proteome database is kept as a reference background proteome, and a library was prepared using the MS2 data obtained from the label-free quantitation (LFQ) analysis. UniProt ID of the proteins (GH, TSHB, AVP, OXT, POMC, POU1F1 [pituitary-specific positive transcription factor 1], NPTX2 [neuronal pentraxin-2], PCOLCE2 [procollagen C-endopeptidase enhancer-2], HSD17B7, etc.) is used as an input list. Unique peptide sequences with 8 to 20 amino acids and 0 missed cleavage of the mentioned proteins were exported as an isolation list and fed into the PRM method, as discussed earlier. Furthermore, the PRM raw files were analyzed using Skyline by considering the peak areas of the peptides.

### Identification, Structure, and Function of uPE1 Proteins

The list of DEPs was searched for PE1 protein without any function and structure in NeXtProt using its API (https://api.nextprot.org/). The output list was further searched in UPEFinder (https://upefinder.proteored.org/) and PHAROS (https://pharos.nih.gov/) to crosscheck the uPE1 status. Proteins that passed two of the three criteria (PubMed score <5, antibody count <50, and gene reference into functions [geneRIFs] <3) were included in the study. Structural and functional prediction of proteins was done using the Human Proteome Structure and Function workflow (21, 22). In short, the proteins were first blasted in UniProt (https://www.uniprot.org/blast/) to identify homologous proteins using default settings. The peptides identified from proteins were further checked for uniqueness using Peptide uniqueness checker (https://www.nextprot.org/tools/peptide-uniqueness-checker). Proteins were then submitted to the i-TASSER server and AlphaFold (https://alphafold.ebi.ac.uk/) (23) to predict structure. Protein Data Bank (PDB) structure obtained was further submitted into COFACTOR (https://zhanggroup.org/COFACTOR/) (24) for function prediction. The predicted structure and function were further curated using different scores generated from i-TASSER (https://zhanggroup.org/I-TASSER/) (25), AlphaFold, and COFACTOR. The structure selection for i-TASSER is based on C-Score, RMSD-Score, and TM-Score, whereas the functional prediction of COFACTOR is based on C-ScoreGO (22). Furthermore, the functional characterization of the uPE1 proteins has been carried out in ProteomeHD tools (26) with a query cutoff score of 0.99.

## Results and Discussion

In this section, we will discuss a comprehensive proteomic analysis of the pituitary gland's matched anterior and posterior lobes that led to identifying pituitary hormone proteins specific to their lobes with their target verification and chromosome map. Here in this section, we will further emphasize the role of pituitary-enriched protein, hormones, and their target verification, lobe-specific major interactors and pathways identified, and S100 as a tumor marker, calcium homeostasis in the posterior lobe, and uPE1 proteins identified.

### Matched Pituitary Anterior and Posterior Lobe–Based Proteome Profiling

**Fig. 1:**
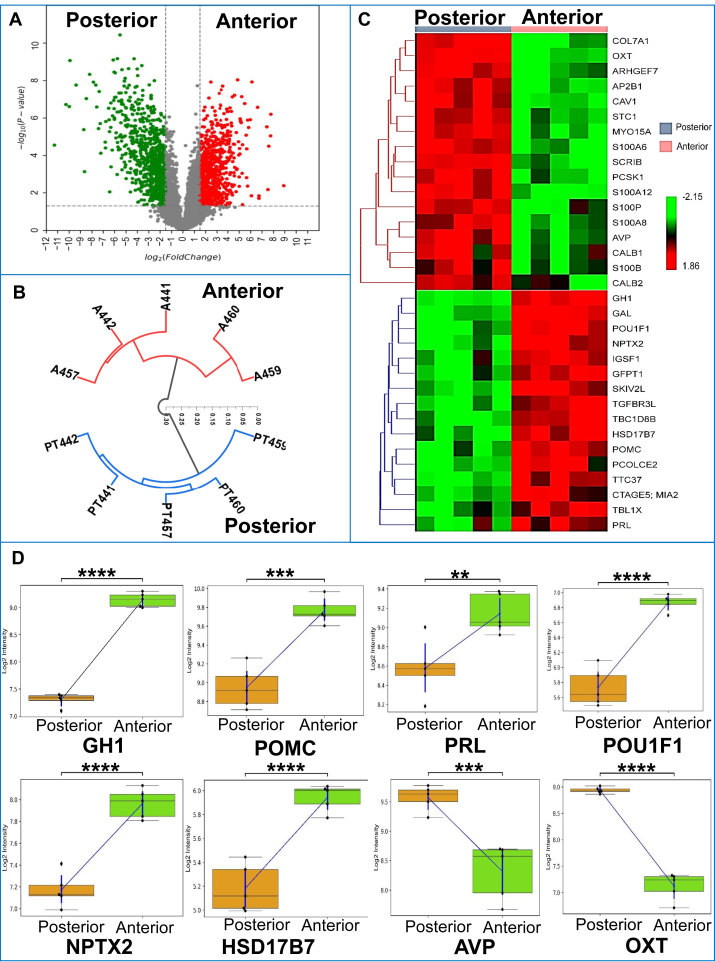
**Statistical analysis of anterior and posterior lobes of the pituitary.***A*, volcano plot of anterior *versus* posterior pituitary proteins. *B*, hierarchical clustering of anterior and posterior samples. *C*, clustered heat map of top 33 proteins from the anterior and posterior lobes. *D*, box plot representative of eight proteins is showing upregulation and downregulation in the anterior and posterior pituitary.

LFQ of matched pituitary lobes identified around 4090 proteins (with isoforms) (supplemental Table S3), 43,880 peptides (supplemental Table S4), and 666,270 PSMs (supplemental Table S5). The log10 normalized abundances obtained from PD2.4 showed a Pearson correlation coefficient greater than 0.6 (supplemental Fig. S2*A*). Of 4090 proteins identified, 101 and 77 proteins were found exclusive to the anterior and posterior lobes (supplemental Fig. S2*B*). Of 2174 peptide groups, 1763 and 391 showed phospho and Met-loss+ acetyl (protein N-term) modification exclusively (supplemental Fig. S2*C*). Around 79.97% of PSMs showed 0 missed cleavages (supplemental Fig. S2*D*). These 4090 proteins were further mapped with KEGG, WikiPathways (https://www.wikipathways.org/index.php/WikiPathways), and Reactome (supplemental Fig. S2*E*) integrated into PD2.4 to understand and comprehend their biological processes, cellular components, and molecular functions. In the biological processes, around 2642 (20.57%) and 2535 (19.73%) proteins were mapped to metabolic process and regulation of biological process (supplemental Table S6 and supplemental Fig. S3*A*). In molecular function, around 3231 (34.46%) and 1777 (18.95%) proteins had protein binding and catalytic activity (supplemental Table S7 and supplemental Fig. S3*B*). In cellular components, around 2230 (18.62%) and 1943 (16.22%) proteins were found in the membrane and cytoplasm (supplemental Table S8 and supplemental Fig. S3*C*). A list of 1446 DEPs with a *p* ≤ 0.05 and log2FC >±1.5 (supplemental Table S9) (Fig. 1) has been further taken forward for the comparative analysis.

### Chromosome Map of Pituitary Proteome

The PD2.4 identified proteins have been further mapped with the NeXtProt 2021 release to curate the chromosome information to extend the goal of the chromosome-centric HPP. The chromosome-wise protein map of the human pituitary was further plotted, showing that many entities were mapped into chromosomes 1, 2, and 11 (supplemental Table S3 and supplemental Fig. S4).

### Comparative Analysis of Pituitary-Enriched and Hormonal Proteins

**Fig. 2:**
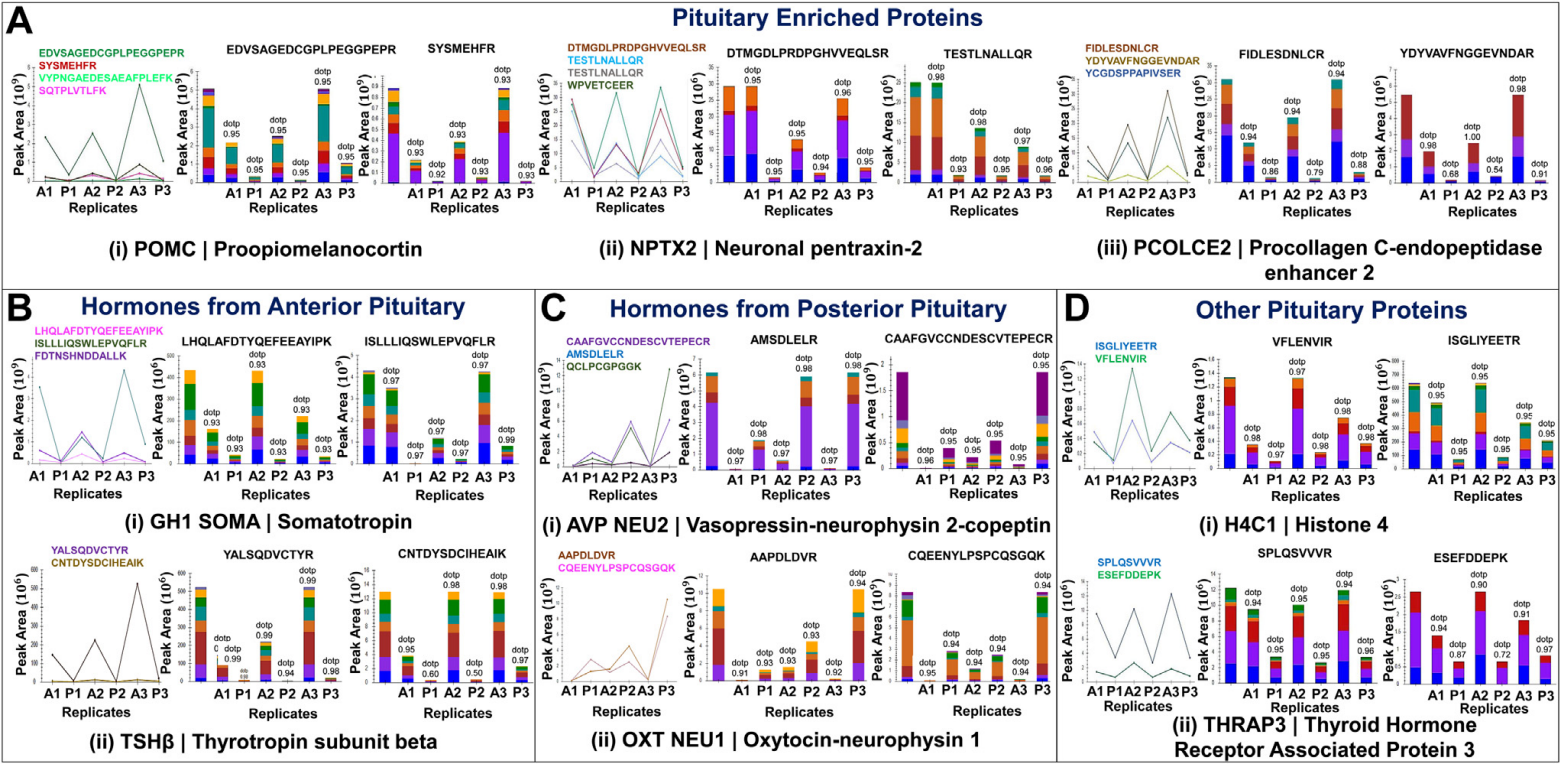
**Target verification of pituitary proteins.***A*, showing peak area comparison of peptides for pituitary-enriched proteins (POMC, NPTX2, and PCOLCE2). *B*, showing peak area comparison of peptides for hormones GH1, TSHβ, AVP, and OXT mapped to specific lobe. *C*, verification of H4, THRAP3, and GAS6 with PRM in both anterior and posterior matched pair. PRM, parallel reaction monitoring. *D.* Other pituitary proteins H4C1 and THRAP3 showing significantly higher expression in the anterior pituitary.

DEPs identified were mapped with the neuroendocrine and brain regions curated from Brain Atlas of Human Protein Atlas (version 20.1), including the adrenal gland, ovary, testis, pancreas, parathyroid gland, midbrain, olfactory region, amygdala, basal ganglia, cerebellum, cerebral cortex, hippocampal formation, hypothalamus, thalamus, pons, and medulla. The comparison aims to identify the enriched proteins with high specificity to pituitary gland in comparison to other brain and endocrine regions based on the normalized RNA expression levels. A list of 26 proteins with high RNA expression in the pituitary was identified (Table 1). Of these 26 proteins, 6 and 20 proteins showed overexpression in the pituitary gland's posterior and anterior lobes, respectively. Protein POU1F1 transcription factor plays a crucial role in the differentiation of various pituitary cell phenotypes, POMC secreted from corticotroph and produces corticotrophin (ACTH); PRL secreted from lactotrophs and key for lactation; GAL (galanin peptides) that plays a crucial role in the neural control of GH, PRL, and ACTH secretion (27); NPTX2 synaptic proteins involved in cell plasticity (28); IGSF1 (immunoglobulin superfamily member-1), which is highly expressed in pituitary cells having POU1F1 lineage (29); PCOLCE2, which plays a crucial role in collagen formation; TGFBR3L (transforming growth factor-beta receptor type 3-like protein), which is gonadotroph specific and expressing in cells having high expression of FSH and LH (30), were highly pituitary specific because of their elevated RNA expression and showed high LFQ protein abundance in the anterior lobe. Whereas stanniocalcin-1 (STC1) (9) regulatory endocrine hormone that maintains the calcium balance, MYO15A (unconventional myosin-XV), having actin-binding and calmodulin-binding properties, showed high pituitary specificity and higher expression of proteins in the posterior lobe. In addition, proteins POU1F1, POMC, PCOLCE2, and NPTX2 (supplemental Table S15, Figs. 2 and S7) have been verified for their prominent protein expression in the anterior lobe using PRM.

**Table 1:** Represents the top 26 pituitary-enriched proteins and their RNA expression in other neuroendocrine glands and brain regions

UniProt	Evidence	Gene symbol	Chromosome	Description	Tissue RNA—adrenal gland	Tissue RNA—ovary	Tissue RNA—pancreas	Tissue RNA—parathyroid gland	Tissue RNA—pituitary gland	Tissue RNA—testis	Tissue RNA—midbrain	Tissue RNA—olfactory region	Brain RNA—amygdala	Brain RNA—basal ganglia	Brain RNA—cerebellum	Brain RNA—cerebral cortex	Brain RNA—hippocampal formation	Brain RNA—hypothalamus	Brain RNA—pons and medulla	Brain RNA—thalamus	AntvsPos_log2(FC)	*p*	FDR	Biological process	Cellular component	Molecular function
Q96ST8	PE1	CEP89	19	Centrosomal protein of 89 kDa	9.5	17.1	9	11.3	20.2	8.4	7.9	8.4	8.5	12.1	16.4	14.4	8.1	6.4	9.1	8.4	−4.04123763386833	0.000014258	0.00018121	Cell communication; cell organization and biogenesis	Cytoplasm; cytoskeleton; cytosol; membrane; mitochondrion	Protein binding
Q9UKN7	PE1	MYO15A	17	Unconventional myosin-XV	0	3	0.5	2.2	46.1	7.4	0.5	0.5	0.5	0.7	2.3	1.5	0.5	1.2	0.7	0.5	−3.99125469952828	0.000019701	0.00022875	Response to stimulus	Cytoplasm; cytoskeleton	Catalytic activity; motor activity; nucleotide binding; protein binding
P52823	PE1	STC1	8	Stanniocalcin-1	2	7.1	9.5	7.7	19.6	4.6	3.5	0	3.5	8.3	2.9	15.9	1.6	3.4	11	3.2	−3.79742625912772	0.00028265	0.0014693	Cell organization and biogenesis; cell proliferation; cellular homeostasis; regulation of biological process; response to stimulus	Cytoplasm; extracellular; membrane; nucleus	Protein binding
Q8TDL5	PE1	BPIFB1	20	BPI fold–containing family B member 1	0	0.1	0.1	0	4.7	1.1	0.1	0.1	0.2	0.2	0.1	0.2	0.2	0	0.1	0.1	−2.69828135982236	0.00015593	0.00095042	Defense response; regulation of biological process; response to stimulus	Extracellular	
P03973	PE1	SLPI	20	Antileukoproteinase	0.4	5.5	5.4	0.1	25.9	7.1	0.3	0.4	0	0	0.1	0.2	0	0	4.7	0	−2.43763722959359	0.00041857	0.002005	Defense response; regulation of biological process; response to stimulus; transport	Extracellular; Golgi; organelle lumen	DNA binding; enzyme regulator activity; protein binding; RNA binding
P35558	PE1	PCK1	20	Phosphoenolpyruvate carboxykinase, cytosolic [GTP]	0.1	0.4	0.2	0	5	0.2	0.2	0.2	0.4	0.4	0.2	0.6	0.3	0.2	0.5	0.2	−2.23774388995667	0.00042794	0.0020356	Cell communication; metabolic process; regulation of biological process; response to stimulus	Cytoplasm; cytosol	Catalytic activity; metal ion binding; nucleotide binding
Q06210	PE1	GFPT1	2	Glutamine-fructose-6-phosphate aminotransferase	11.4	11.1	34.9	13.9	39.6	13.9	6.5	5.7	6.4	7.8	7.8	10.1	6	9.8	7	4.8	1.79542397969551	0.00026083	0.001381	Metabolic process; regulation of biological process	Cytosol	Catalytic activity
P01236	PE1	PRL	6	Prolactin	0	0	0	0	1739.5	1.3	0	0	0	0.1	0	0.1	0	0	0.6	0	1.87926403311717	0.0077107	0.018966	Cell proliferation; metabolic process; regulation of biological process; response to stimulus; transport	Extracellular; organelle lumen	Protein binding
Q9UKZ9	PE1	PCOLCE2	3	Procollagen C-endopeptidase enhancer 2	1.2	2.5	0.6	0.1	13.8	1.9	0.6	0.7	1	0.5	0.3	0.7	1.4	1.2	0.4	0.3	1.91961397809188	0.00037768	0.0018467	Regulation of biological process	Extracellular	Enzyme regulator activity; protein binding
Q99959	PE1	PKP2	12	Plakophilin-2	0.7	9.5	5.4	9.6	14.7	3.4	0.7	2	1.4	5.2	2.2	1.8	3.9	0.9	3.8	0.4	1.91990874213475	0.00010131	0.00071611	Cell communication; cell death; cell organization and biogenesis; regulation of biological process	Cytoskeleton; membrane; nucleus	Protein binding; structural molecule activity
P29508	PE1	SERPINB3	18	Serpin B3	0	0.2	0.2	0	2.4	0.2	0.2	0.2	0.2	0.2	0.2	0.2	0.2	0	0.2	0.2	1.98402025992868	0.0040443	0.011427	Cell communication; regulation of biological process; transport	Cytoplasm; extracellular; nucleus; organelle lumen	Enzyme regulator activity; protein binding; receptor activity
P53420	PE1	COL4A4	2	Collagen alpha-4(IV) chain	8.8	3.2	6.6	0.5	15.3	1.9	2.4	0	1.2	2.4	1	1.8	1.4	1.5	7.4	1.5	2.03598809996206	0.0015384	0.0053435	Cell organization and biogenesis; metabolic process	Extracellular; organelle lumen	Structural molecule activity
Q8N6C5	PE1	IGSF1	X	Immunoglobulin superfamily member 1	11.9	1	1.7	0	138.7	11.8	3.8	1.9	1.4	5.3	0.6	2.3	3.3	15.4	4.7	1.9	2.52137506832756	0.0011821	0.0043496	Regulation of biological process;response to stimulus	Extracellular; membrane	Protein binding; receptor activity; signal transducer activity
P22466	PE1	GAL	11	Galanin peptides	0.2	0.2	2.9	0	149.9	0.5	0.2	0.6	0.1	0.5	0.6	0.7	1.3	18.8	2.2	0.4	2.571348083041	0.0000035863	0.000072609	Defense response; regulation of biological process; response to stimulus; transport	Cytoplasm; extracellular; Golgi	Protein binding;receptor activity; signal transducer activity
P47972	PE1	NPTX2	7	Neuronal pentraxin-2	24	1.5	19.5	0.8	71.6	20.7	9.4	2.2	7.1	8.4	0.2	24.6	9.2	11	7	5	2.62999536364986	0.000026736	0.00027906	Cell communication; response to stimulus	Extracellular	Metal ion binding
O95965	PE1	ITGBL1	13	Integrin beta-like protein 1	6.3	3.6	5.2	3.4	18.8	3.8	4	4	1.2	1.7	8.3	1.6	2.7	2.2	5.9	1.6	2.64189380885636	0.000068474	0.0005463	Regulation of biological process; response to stimulus	Extracellular; membrane	
P01189	PE1	POMC	2	Pro-opiomelanocortin	3.2	0.3	22.2	0	811.1	2.9	0.3	0.3	0.3	0.3	0.3	0.3	0.3	1.8	0.3	0.3	2.71463462120455	0.00011201	0.00076175	Cell communication; metabolic process; regulation of biological process; response to stimulus	Cytoplasm; extracellular; organelle lumen	Protein binding
O60907	PE1	TBL1X	X	F-box-like/WD repeat–containing protein TBL1X	7.1	13.8	11.3	21	24.3	9.9	7.6	6	10.1	12.3	3.3	8.1	8.6	9	7.5	8.5	2.84618076568573	0.0015739	0.0054337	Cell organization and biogenesis; metabolic process; regulation of biological process; response to stimulus	Nucleus	DNA binding; protein binding
P22413	PE1	ENPP1	6	Ectonucleotide pyrophosphatase/phosphodiesterase family member 1	2.1	7.3	17.5	22.2	27	4.6	1.1	0	0.5	0.5	0.6	0.4	0.5	0.7	0.8	0.3	3.40797155935889	0.000000031816	0.0000041351	Cellular homeostasis; metabolic process; regulation of biological process; response to stimulus; transport	Cell surface; extracellular; membrane	Catalytic activity; metal ion binding; nucleotide binding; protein binding; receptor activity
Q5VV41	PE1	ARHGEF16	1	Rho guanine nucleotide exchange factor 16	0.1	0.9	7	4.8	7.7	2.3	1.3	0.5	0.5	1	1.1	0.8	1.1	0.9	2.2	0.7	3.48830957897704	0.011963	0.027062	Cellular component movement; regulation of biological process; response to stimulus	Cytoplasm; cytosol	Protein binding
Q0IIM8	PE1	TBC1D8B	X	TBC1 domain family member 8B	24.5	7.2	10.7	10	28.6	7.6	6.8	3.3	4.6	7.9	1.2	7.9	4.2	2.5	9	7.6	3.51683755762071	0.000017595	0.00021054	Regulation of biological process; transport		Enzyme regulator activity; metal ion binding; protein binding
P28069	PE1	POU1F1	3	Pituitary-specific positive transcription factor 1	0.7	0	0	0	138.8	0.2	0	0	0	0	0	0	0	0	0	0	3.7697812409231	0.000015713	0.000196	Cell differentiation;metabolic process; regulation of biological process; transport	Nucleus	DNA binding; protein binding
Q5TF21	PE1	SOGA3	6	Protein SOGA3	2.8	3.3	1.7	0.2	11.9	1.8	6.7	5	5	5.3	11.4	8.2	4.2	4.5	7.9	4.3	4.40863462174743	0.0046771	0.012681	Metabolic process; regulation of biological process	Membrane	
Q96ET8	NA	FAM18B2; TVP23C; FAM18B2-CDRT4; TVP23C-CDRT4	17	Golgi apparatus membrane protein TVP23 homolog C	5.8	10	6.2	5.4	19	8.8	4.2	6.6	4.4	6.1	15	9.1	4.2	4.2	10.2	3.1	4.58679767886429	0.000025768	0.00027321	Transport	Membrane	
H3BV60	PE1	TGFBR3L	19	Transforming growth factor-beta receptor type 3-like protein	2	0.3	0.3	0.9	56.6	0.5	0.5	1.7	1.6	2.8	1.9	6.4	0.7	0.8	1.9	0	5.06884603780208	0.000083729	0.00062587		Membrane	
Q12802	PE1	AKAP13	15	A-kinase anchor protein 13	11.1	20	26.4	8	40.5	15	9	5.5	7.2	9.3	1.9	7.8	8.8	6.1	12.4	7.1	5.18450386511795	0.0000017939	0.000044836	Cell differentiation;metabolic process; regulation of biological process; response to stimulus; transport	Cytoplasm; cytoskeleton; cytosol; membrane; nucleus	Catalytic activity; metal ion binding; protein binding; signal transducer activity; structural molecule activity

Our pituitary proteome map also resulted in the identification and target verification of hormonal proteins like GH (somatotropin) and TSHβ (thyrotropin subunit beta) to be highly specific to the anterior lobe and AVP (vasopressin neurophysin 2-copeptin) and OXT specific to the posterior lobe (supplemental Table S9 and Fig. 1). Both GH, TSHβ and AVP, OXT (supplemental Table S15 and, Figs. 2 and S7) were verified with PRM.

### Anterior and Posterior Lobe Pathway Analysis, Interactor Study, and their Lobe Specificity

Pathway analysis of 1446 DEPs (687 upregulated in anterior and 759 upregulated in posterior) (supplemental Table S9) was further analyzed in Reactome to identify the pathways specific to each lobes (supplemental Table S10). Transcription, translation, post-translational modification, vesicular trafficking, and so on are the basic cellular processes related to the pituitary gland's secretory nature.

**Fig. 3:**
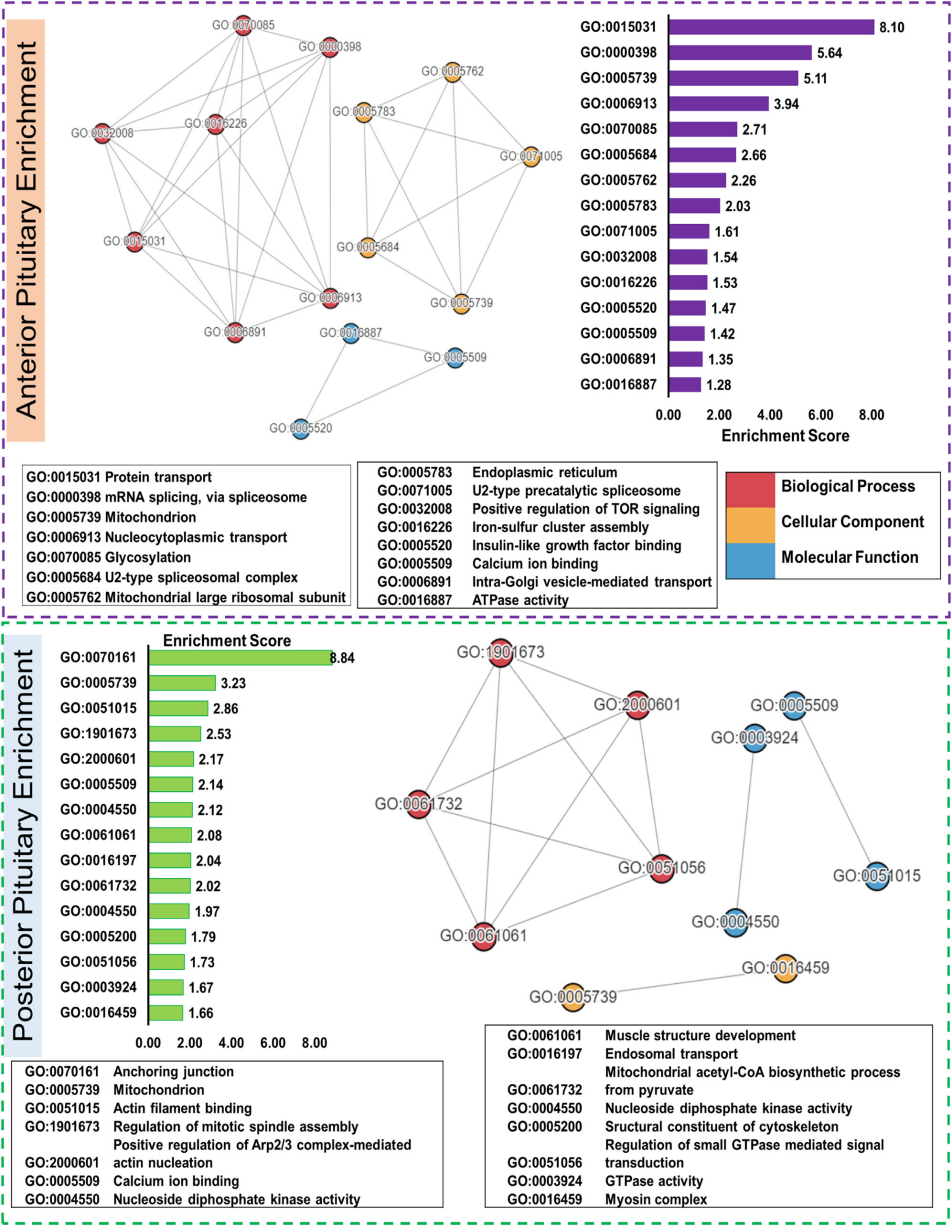
**Functional clustering and enrichment of differentially expressed protein in anterior and posterior lobes of pituitary**.

**Fig. 4:**
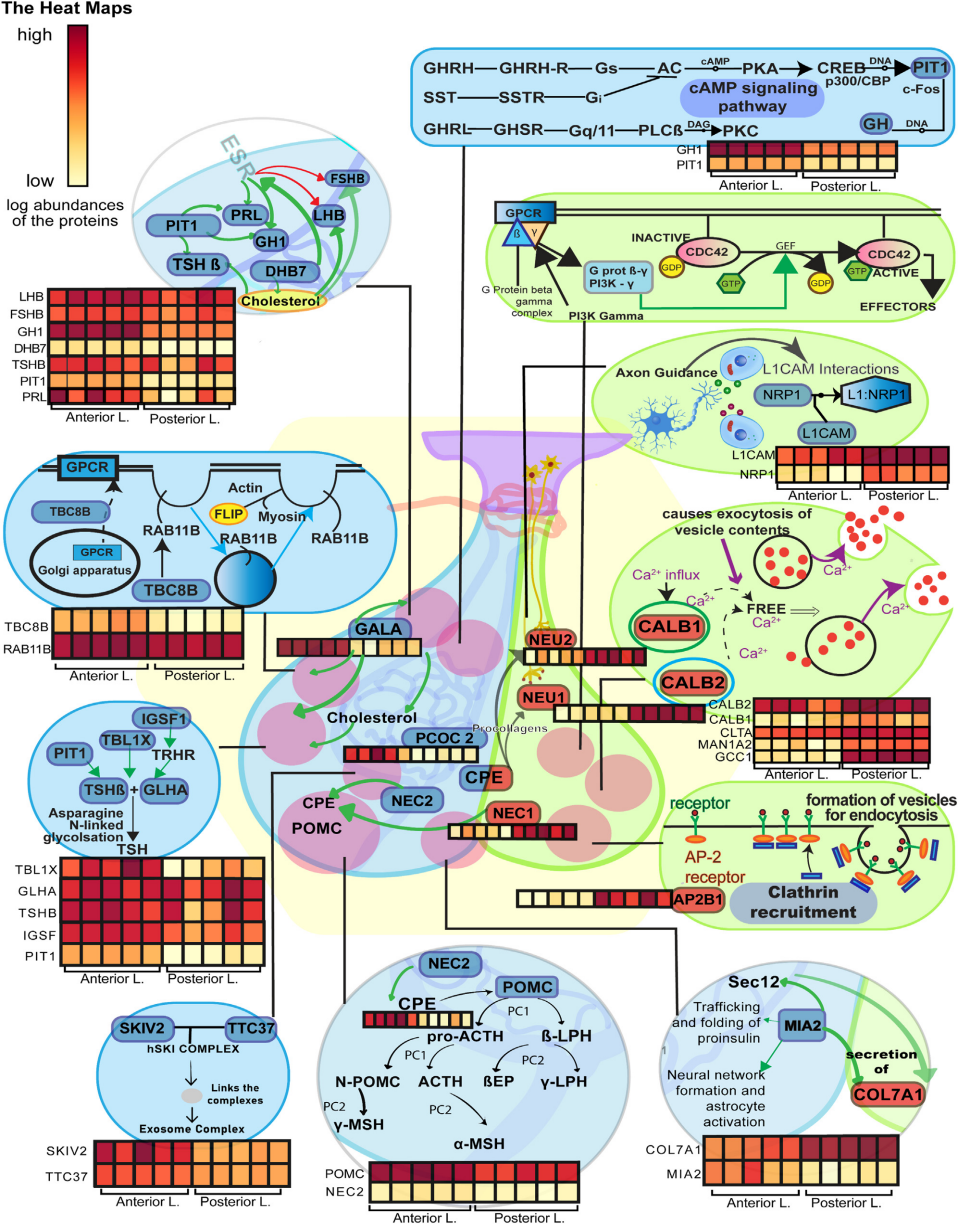
**The figure shows the significant proteins and their pathways in respective lobes, along with protein heatmaps.***Black arrows* denote that a protein is a part of the following entity, *green arrows* denote upregulation, and *red arrows* denote downregulation. GH, LHB, FSHB, PRL, TSHB, POMC, and GLHA are anterior lobe hormone constituent proteins, whereas AVP, OXT (products of NEU1, NEU2) are posterior lobe hormones. NEC2, CPE, PCSK1, and PCSK2 are involved in the sorting and cleavage of hormonal proteins, whereas Pit-1, TBL1X, IGSF1, and GALA regulate transcription and production. MIA2, CALB1, CALB2, TBC1D8B, and AP2B1 are concerned with protein trafficking, exocytosis, endocytosis, and vesicular transport. Some noteworthy posterior lobe–specific pathways regarding Rho GTPase downstream signaling, clathrin recruitment, neural cell development, and maintenance have been illustrated.

As most of the hormone synthesis occurs abundantly in the anterior lobe, it showed eminently high expression in transcription, translation, and post-translational modification in the anterior lobe. Pathways concerned with creating the final mRNA product at the transcriptional (pre-mRNA synthesis) or post-transcriptional level (splicing, capping, etc.) were found to be of high significance in the anterior pituitary. Pathways include processing of capped intron–containing pre-mRNA (R-HSA-72203), mRNA splicing (R-HSA-72163), transport of mature mRNA derived from an intron-containing transcript (R-HSA-159236), transport of mature transcript to cytoplasm (R-HSA-72202), mRNA splicing (R-HSA-72172), metabolism of RNA (R-HSA-8953854), mRNA 3′-end processing (R-HSA-72187), and metabolism of proteins (R-HSA-392499) highly abundant in the anterior pituitary. The GO functional clustering analysis output has also been found to match with the pathway analysis, where protein transport (GO:0015031), mRNA splicing *via* spliceosome (GO:0000398), U2-type spliceosomal complex (GO:0005684), U2-type precatalytic spliceosome (GO:0071005), intra-Golgi vesicle-mediated transport (GO:0006891) were found to be significant in anterior lobe having enrichment score ≥1.5 (Figs. 3 and 4, supplemental Tables S10 and S11). Proteins like TBL1X (transducin beta-like protein 1), IGSF1, and TGFBR3L were highly significant in the pathways and upregulated in the anterior pituitary proteome. TBL1X maintains basal activation and repression of TSH at the nuclear level (31). IGSF1 is a gene associated with congenital central hypothyroidism. Joustra *et al.* (32) studied patients with IGSF1 deficiency, which showed decreased pulsatile secretion of TSH, abnormal PRL secretion, and over a 200% rise in FSH levels. TGFBR3L in human gonadotropes (30) correlates with FSH and LH and negatively correlates with estrogen receptors, which subsequently play a role in gonadotrope development and function. Translation of proteins (R-HSA-72766) was also significantly upregulated in the anterior lobe, which explains higher hormone secretion levels in the anterior lobe. Proteins POU1F1, TSHβ, POMC, PLCB4, PRKACB, and SOMA are involved in transcription and hormonal biosynthesis and were significantly upregulated in the anterior lobe, whereas OXT and AVP (supplemental Tables S9–S11 and Figs. 3 and 4) were upregulated in the posterior lobe. Proteins POU1F1, TSHβ, and POMC were validated (Figs. 2 and S7 and supplemental Table S15) using PRM.

Post-translational modifications such as SUMOylation of SUMOylation proteins (R-HSA-4085377), SUMOylation of chromatin organization proteins (R-HSA-4551638), SUMOylation of ubiquitinylation proteins (R-HSA-3232142), SUMOylation of DNA replication proteins (R-HSA-4615885), SUMOylation of RNA-binding proteins (R-HSA-4570464), SUMOylation of DNA damage response and repair proteins (R-HSA-3108214), SUMOylation (R-HSA-2990846), and asparagine-N-linked glycosylation (R-HSA-446203) and glycosylation (GO:0070085) were found to be highly significant in the anterior lobe (supplemental Tables S9–S11 and Figs. 3 and 4). SUMOylation is required for various post-translational modifications of proteins such as TSHβ, PTTG1 (pituitary tumor–transforming 1), histone acetylation, and plays a crucial role in nuclear program modification, development, cholesterol homeostasis, and adaptation to stressful conditions (33, 34, 35). Sujie *et al.* (33) showed sumoylation of TSHβ plays a crucial role in thyrotropin regulation and TSH release. Histone sumoylation, acetylation, and deacetylation play a crucial role in transcription; higher expression of HDAC7 (histone deacetylase 7), KDM1A (lysine-specific histone demethylase 1A), and HDAC1 (histone deacetylase 1) is seen in the anterior lobe (supplemental Table S9). It may be involved in the gene expression of pituitary-enriched proteins and hormones. H4 protein is shown to have higher expression in the anterior lobe and validated using PRM (Figs. 2 and S7, and supplemental Table S15), resulting from histone modification in the anterior lobe. Asparagine-N-linked glycosylation plays a crucial role in protein folding, solubility, stability, specificity, and function of the protein (36). Proteins TSH, FSH, and LH (supplemental Tables S9, S10 and supplemental Fig. S5) are N-glycosylated, which are critical for hormonal activity; furthermore, in the absence of N-linked oligosaccharides, these hormones behave as their antagonist (37). Thus, we can conclude that the anterior lobe is more active in protein synthesis and metabolism.

While the pathways of vesicular transport (R-HSA-5653656) hold higher significance in the posterior lobe, it is worth that several proteins involving MIA2 or cTAGE5 (melanoma inhibitory activity protein 2), TVP23C (TVP23 homolog C), and TBC1D8B (TBC-1 domain family member 8B) showed higher expression in anterior pituitary proteome (supplemental Table S9). MIA2 is involved in the secretion of collagen VII and large cargo proteins from the endoplasmic reticulum (38). Recent studies showed MIA2 also plays an essential role in the trafficking and folding (39) of proinsulin; its absence can result in dysregulation of insulin biogenesis. MIA2 (supplemental Table S9 and Fig. 4) also plays a role in neural network formation and astrocyte activation (40).

Pathways of nervous system development (R-HSA-9675108) and axon guidance (R-HSA-422475) were significant in the posterior lobe. Protein NRP1 (neuropilin-1) is responsible for neurovascular maintenance through positive feedback of vascular endothelial growth factor (41) and has previously shown to be abundant in pituicytes (42); L1CAM (neural cell adhesion molecule L1) deficiency has been related with hypoplastic posterior pituitary (43) and plays a crucial role in brain growth and neuronal migration (44); PLXNC1 (plexin-C1) was found to be highly abundant in the posterior pituitary proteome data (supplemental Tables S9 and S10). GO functional clustering study identified anchoring junction (GO:0070161), actin filament binding (GO:0051015), and myosin complex (GO:0016459) to be significant in anterior lobe having enrichment score ≥1.5 (Figs. 3, 4 and supplemental Tables S10 and S11).

Pathways of membrane trafficking (R-HSA-199991), vesicle-mediated trafficking (R-HSA-5653656), endosomal transport (GO:0016197), regulation of small GTPase-mediated signal transduction (GO:0005200), and GTPase activity (GO:0003924) were also upregulated in the posterior pituitary. Proteins RAB3GAP2 (Rab3 GTPase-activating protein noncatalytic subunit), GCC1 (GRIP and coiled-coil domain–containing protein), MAN1A2 (mannosyl-oligosaccharide 1,2alpha-mannosidase IB), SNAP91 (clathrin [CLAT] coat assembly protein AP180), CLTA (CLAT light chain A), and AVP mapped in pathways were upregulated in posterior pituitary (supplemental Tables S9–S11). Multiple minor pathways within the larger domain of Rho GTPase cycles (R-HSA-9012999), signaling by Rho GTPases (R-HSA-194315) and CLAT-mediated endocytosis (R-HSA-8856828), were found to be also upregulated in the posterior pituitary. Protein AVP was commonly found in CLAT-mediated endocytosis, membrane trafficking, vesicular transport, R-HSA-432040 (vasopressin regulates renal water homeostasis *via* aquaporins), and aquaporin-mediated transport (R-HSA-445717). Protein AVP (Figs. 2 and S7) was validated using PRM.

### Protein Interaction Study of Anterior and Posterior Lobes

Protein–protein interaction analysis of the anterior lobe led to the identification of protein HDAC1 protein hub, which has been studied in the nonfunctional pituitary adenomas and Cushing's (45, 46), behaves as tag for epigenetic repression, and play an important role in transcriptional regulation, cell cycle progression, and developmental events. Protein HDAC1 and their interactor KDMA1, which functions as specific tag for epigenetic transcriptional activation and regulates pituitary gland development (47), proteins MTA1 and MTA2 (metastasis-associated protein, functioning as transcriptional coregulator), protein TBL1X plays an essential role in transcription activation mediated by nuclear receptors, and its mutation leads to hypothyroidism, protein FKBP3 (FK506-binding protein 3, which regulates protein folding and trafficking and HDAC7 along with MSH6) (supplemental Fig. S6 and supplemental Table S13) was found to be significant in histone deacetylase protein hub.

Anterior lobe being transcriptionally and translationally active proteins, such as NCBP1 (nuclear cap–binding protein subunit 1, which regulates pre-mRNA splicing, translation regulation, and mRNA export) along with their interactors HNRNPR (heterogeneous nuclear ribonucleoprotein R, which regulates processing of precursor mRNA in the nucleus), small nuclear ribonucleoprotein such as (SNRNP40, SNRPD1, which catalyzes the removal of introns from premessenger RNAs), U6 snRNA-associated Sm-like protein (LSM2, LSM4, LSM7, involved in pre-mRNA splicing), splicing factor 3A and 3B subunit (SF3A1, SF3A2, SF3B2, SF3B5), serine/arginine-rich splicing factor (SRSF3, SRSF7, which specifically promotes exon inclusion during alternative splicing), pre-mRNA-splicing factor ISY1, TSN (translin), AGO argonaute protein (AGO1, AGO2, AGO3, which play crucial role in functioning of small RNA), tRNA ligase, and tRNA synthetase (such as EPRS, LARS, VARS, IARS, FARSA, FARSB) were found to be significantly clustered (supplemental Fig. S6 and supplemental Table S13) in protein hub responsible for transcription and translation. Protein hub belonging to phosphatases play a crucial role in the development of the brain (48); protein phosphatase (PPP1CC, PPP1R8, PPP2R4, PPP4R2, and SMEK1 play a crucial role in the regulation of glycogen metabolism, muscle contractility, and protein synthesis) with their interacting hub consisting of protein kinases (PRKACB and PRKAG1), A-kinase anchor proteins (AKAP13 and AKAP9), protein OSBP (oxysterol-binding protein 1, which regulates lipid metabolism), protein ITPR1 (inositol 1,4,5-trisphosphate receptor type 1), Ras GTP-related proteins such as RAB13, RRAGA, RRAGC, and LAMTOR1 (late endosomal/lysosomal adaptor were found to be significantly clustered in protein phosphatase and kinase hub in anterior lobe. Vesicular trafficking plays an important role in the secretion of hormones from the anterior pituitary. Protein GOLGA4 (Golgin subfamily A member 4) protein hub with their interactors coatomer subunit (COPE, COPG2, COPA, COPB2 play essential role for the retrograde Golgi-to-endoplasmic reticulum transport), protein USO1 (general vesicular transport factor p115), and conserved oligomeric Golgi complex (COG1, COG3, COG4, COG5, and COG7) protein hubs (supplemental Fig. S6 and supplemental Table S13) were also highly significantly clustered in vesicular trafficking protein hub in the anterior pituitary.

Posterior lobe of the pituitary is neural ectoderm in origin and maintains water balance, neural development, muscle structure, and calcium homeostasis, mainly because of the secretion of AVP and OXT. Integrin proteins play a significant role in developmental angiogenesis in the pituitary gland (49). Integrin beta/alpha protein hub (ITGB1, ITGB2, ITGB8, ITGA3, and ITGA7) along with their protein interactors IGSF8 (immunoglobulin superfamily member 8), JAM2 (junctional adhesion molecule B), VCAM1 (vascular cell adhesion protein 1), and collagen proteins (COL3A1, COL5A2, and COL7A1) was highly significant in posterior lobes. Protein kinase and phosphorylase protein hub consisting of PTK1 (focal adhesion kinase 1, which regulates axon growth and neuronal cell migration) and phosphoinositide-3-kinase regulatory subunit (PIK3R1, PIK3R4, which plays crucial role in ITGB2 signaling) (supplemental Fig. S6 and supplemental Table S13) were found to be significantly clustered in protein phosphatase and kinase hub in posterior lobe.

Endosomal charged multivesicular sorting proteins (CHMP1A, CHMP2A, CHMP3, CHMP4B, and CHMP6, which play a crucial role in the multivesicular pathway), along with STAMBP (STAM-binding protein), were also significant in the posterior lobe. The posterior pituitary plays a key role in calcium homeostasis, thus regulating water balance, hormonal release, and so on. AP complexes (AP1S2, AP2B1, AP3M2, AP3S1, which mediate both the recruitment of CLAT to membranes and the recognition of sorting signals within the cytosolic tails of transmembrane cargo molecules), calcium–calmodulin-binding proteins and kinases (CAB39L, CAMK2A, and CAMK2D), CLTA (CLAT light chain A), SNX9 (sorting nexin-9, which is involved in endocytosis and intracellular vesicle trafficking), protein SYNJ1 (synaptojanin-1), ITSN1 (intersectin-1), SH3-containing GRB2-like protein (SGIP1 and SH3Gl1, which play vital role in CLAT-mediated endocytosis), and EPN1 (epsin-1, which modifies membrane curvature and facilitates the formation of CLAT-coated invaginations) were also found to be significantly clustered in posterior pituitary. Many other proteins, such as syntaxin proteins (STX1A, STX1B, STXBP5, which mediates Ca^2+^ exocytosis with their protein interactors), vesicle-associated membrane proteins (VAMP3 and VAMP7), SV2A (synaptic vesicle glycoprotein 2, which controls regulated secretion in neural and endocrine cells), and SYT1 (synaptotagmin-1, functioning in the membrane interactions and dendrite formation) were found to be significantly clustered (supplemental Fig. S6 and supplemental Table S13) in posterior lobe.

### S100 as a Tumor Marker, Calcium Homeostasis in the Posterior Lobe

S100 proteins were used as markers for pituitary adenomas (50) and have shown strong immunoreactivity in pituicytomas (51). Our comparative proteomic analysis showed S100s (S100B, S100A6, S100A12, S100A8, and S100P) proteins have high expression in the posterior lobe (supplemental Table S9). S100s are Ca^2+^-binding proteins with multiple functions maintaining intracellular Ca^2+^ homeostasis, signaling (52), and neuroplasticity (53), which play a pivotal role in controlling hormone release from the posterior lobe. Peptidergic nerve endings in the posterior pituitary, responsible for secreting AVP and OXT, release these hormones in response to a Ca^2+^ influx. Cystolic Ca^2+^ buffers regulate free Ca^2+^ levels and control the frequency and pattern of excitation–secretion coupling signals. This mechanism dictates the short-term plasticity of AVP and OXT release (54). Proteomic data showed CALB1 (calbindin) and CALB2 (calretinin), two Ca^2+^ buffers, upregulated in the posterior pituitary (supplemental Table S9). STC1, which showed high specificity for the pituitary, seems to have an inhibitory role in local Ca^2+^ transport (55). The importance of calcium homeostasis is further confirmed by the presence of key proteins involved in Ca^2+^ signaling and mobilization (PRKCA (56), PLCG1, CAMK2A, CAMK2D, CD38, ATP2B3, IGLV3-27, and SYK); pathways of calcium ion binding (GO:0005509) and muscle structure development (GO:0061061) (supplemental Tables S9, S10, S11, Figs. 3 and 4) exhibited high expression in the posterior lobe.

### Structural and Functional Characterization of the uPE1 Proteins

**Fig. 5:**
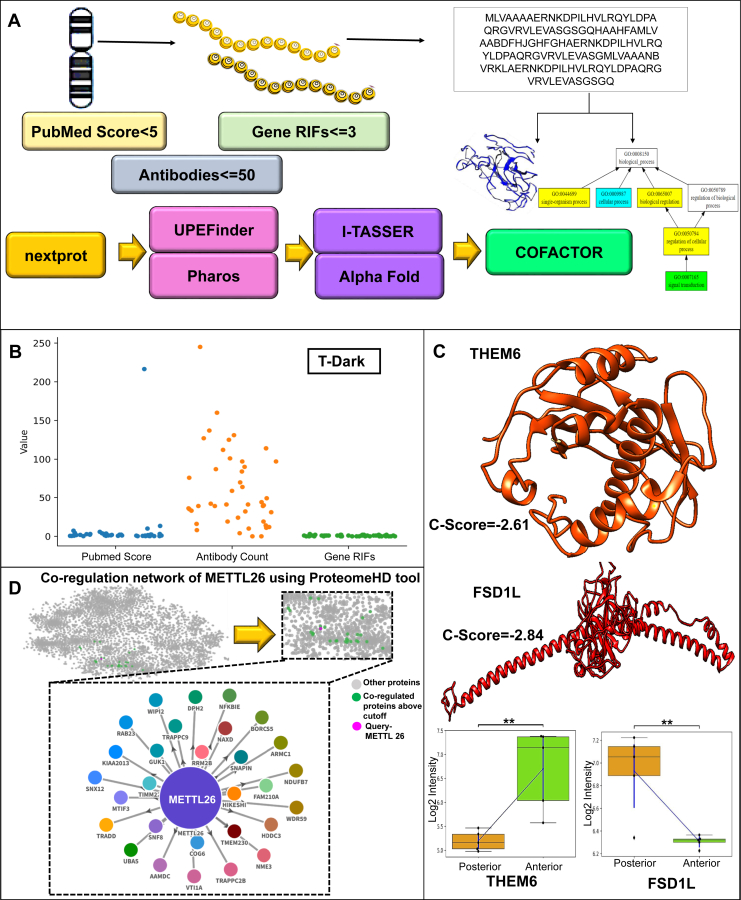
**Structural and functional characterization of uPE1 proteins.***A*, workflow depicting the structural and functional characterization of uPE1 proteins. The workflow includes identification of the proteins having uPE1 status, followed by structural and functional prediction using i-TASSER, AlphaFold, and Chimera. *B*, the value of the PubMed score, antibody count, and geneRIFs of the proteins. *C*, it represents the structure of THEM6 and FSD1L predicted by i-TASSER. *D*, coregulation network of METTL26 using ProteomeHD tool.

Among 1446 DEPs, 44 proteins (supplemental Table S12) matched the uPE1 category according to the NeXtProt database and mapped with their chromosomal information using NeXtProt 2021 release (Fig. 5*A*). Three proteins, THEM6 (mesenchymal stem cell protein DSCD75), METTL26 (methyltransferase-like 26), upregulated in the anterior lobe, and FSD1L (coiled-coil domain–containing protein 10), upregulated in the posterior lobe from pituitary proteome data, were further studied (supplemental Table S9). The functional annotation of the proteins using ProteomeHD with a cutoff score of 0.99 revealed 29 proteins to be coregulated along with METTL26 (supplemental Table S12, Figs. 5 and S5). The coregulated proteins show an enriched purine metabolism pathway (KEGG) with an adjusted *p* value (Bonferroni) of 2.60E-02. Furthermore, 11 of the 29 proteins that are coregulated are found to be a part of protein localization. From this, we can predict the functional association of METTL26 and its role in the anterior pituitary. However, any significant result could not be retrieved for THEM6 and FSD1L using ProteomeHD. Therefore, we followed the structure and prediction workflow as mentioned in the Human Proteome Structure and Function workflow. The PDB structures were obtained from i-TASSER with the best C-Score of −2.61, −0.47, and −2.84, respectively, and AlphaFold having very high confidence (pLDDT >90) (Figs. 5 and S5). PDB models corresponding to the best score were taken forward to carry out characterization. Furthermore, using COFACTOR, protein's function was predicted based on molecular function, biological process, and cellular component. It has predicted the function of the proteins based on molecular function, biological process, and cellular components, as mentioned in supplemental Table S12, Figs. 5 and S5. These are *in silico* predictions and need further validation to conclude their role in the structure and function of normal pituitary (Figs. 5 and S5). The study aimed to provide insights into the workflow of the structural and functional characterization of the uPE1 proteins that are differentially expressed in both the lobes of the pituitary gland. The study on three proteins revealed important insights regarding their molecular function. An extensive follow-up study on all identified uPE1 proteins has the potential to provide further insights regarding their characterization and uPE1 proteins in the pituitary gland as a whole.

### Limitation of Study

The collective study results involve label-free proteome analysis of five matched anterior and posterior lobes of the pituitary because of limitations encountered in acquiring pituitary samples in the best possible condition in which stratification of the intermediate lobe is challenging. The pituitary proteome data are obtained from male individuals and may show different properties from females and thus should be considered with caution during pituitary anterior and posterior lobe lateralization.

## Conclusion

Pituitary being the site of multiple endocrine functions and pituitary adenomas, it is imperative that our understanding of the pituitary function and tumor markers be comprehensive. Our study shows POU1F1, POMC, PCOLCE2, and NPTX2 to be highly pituitary specific compared with other neuroendocrine glands and brain regions, and verification of hormones GH, TSHB and OXT, AVP specific to the respective lobes with PRM. It enhances our understanding of the pathways involved in pituitary functioning and the precise roles that some prominent proteins play within these pathway networks. It also provides a physiochemical basis for substantiating past clinical research on these proteins and their pituitary-related actions. Finally, challenging the accuracy of popularly used pituitary tumor markers, it quantifiably compares the levels of S-100 proteins between the two pituitary lobes to conclude that their expression skews heavily in favor of the posterior pituitary. This pituitary proteomic landscape will help the clinicians and researchers better understand diseases, identifying better prognostic markers and disease diagnostics.

We believe that our first detailed lobe-specific pituitary proteome map will provide researchers with a more comprehensive understanding of pituitary anterior and posterior lobes, identifying proteins expressed in each lobe, biological validation hormones, and their function. The foundation work will further apprehend pituitary physiology and understanding pituitary adenomas (Cushing's, acromegaly, and nonfunctional pituitary adenomas) as reference control will lead to better disease management and accelerate the disease-based investigation. Furthermore, the output of the study supports the goal and objective of the chromosome-centric HPP and strengthens the knowledge-base pillar of HPP.

## Data Availability

LFQ raw data and the PD analyzed output file supporting our research findings are available on ProteomeXchange Consortium (http://proteomecentral.proteomexchange.org) with the identifier PXD030235 (https://www.ebi.ac.uk/pride/archive/projects/PXD030235/private) for ProteomeXchange. The PRM proteomics data are deposited in the Panorama Public and can be accessed through this link: Panorama Public (https://panoramaweb.org/antpstpituitary.url). All supplement data tables are available in Mendeley data under https://doi.org/10.17632/4g8k9hvjcs.1.

## Supplemental data

This article contains supplemental data.

## Conflict of interest

The authors declare no competing interests.
