# Supplementary figures and images for: The First Pituitary Proteome Landscape From Matched Anterior and Posterior Lobes for a Better Understanding of the Pituitary Gland
Source: Mol Cell Proteomics. 2022 Dec 5;22(1):100478. doi: 10.1016/j.mcpro.2022.100478 (PMC9877467; doi:10.1016/j.mcpro.2022.100478)

# Figure S1

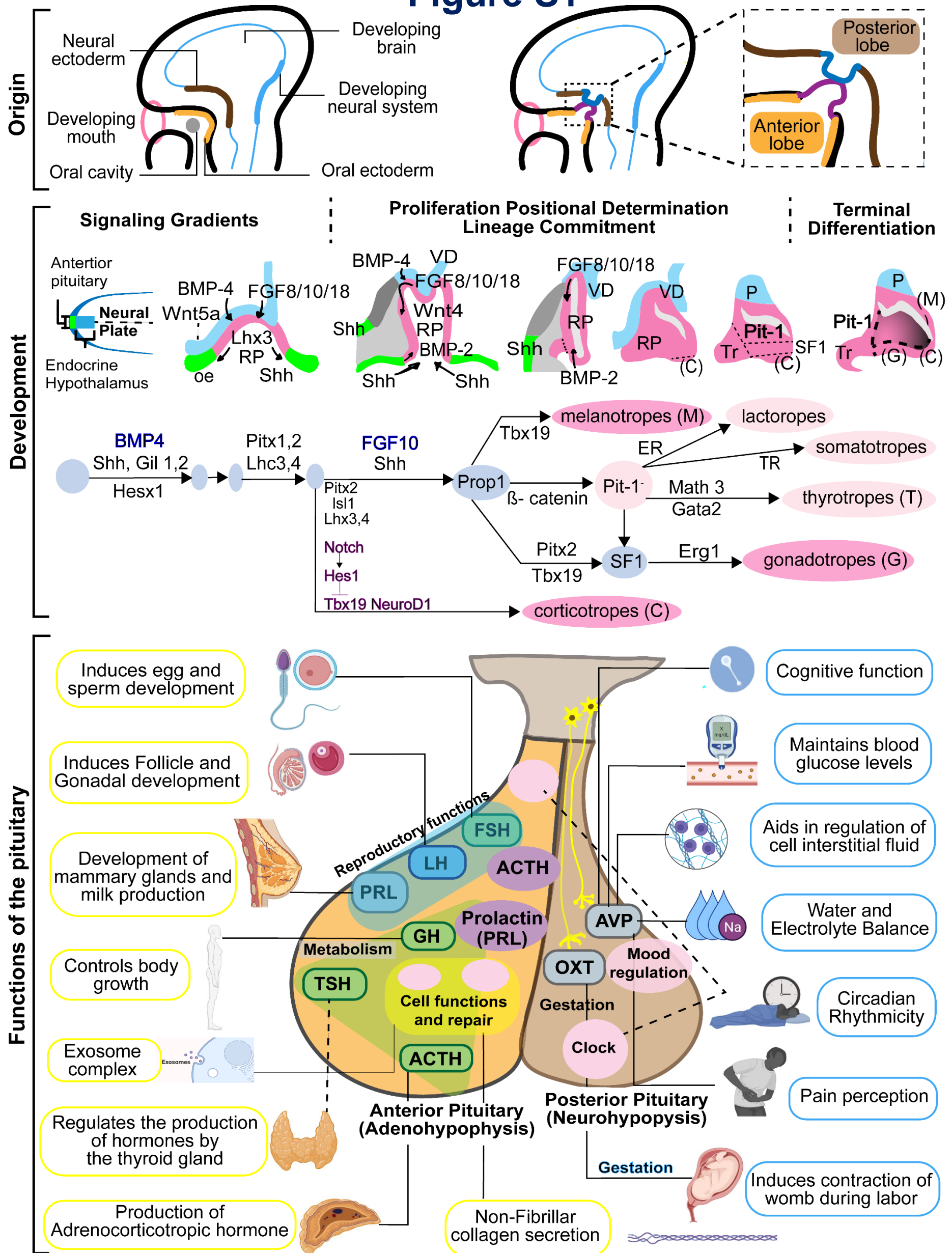

Supplement: Figure S1 [file mmc1.pdf]

Figure S3

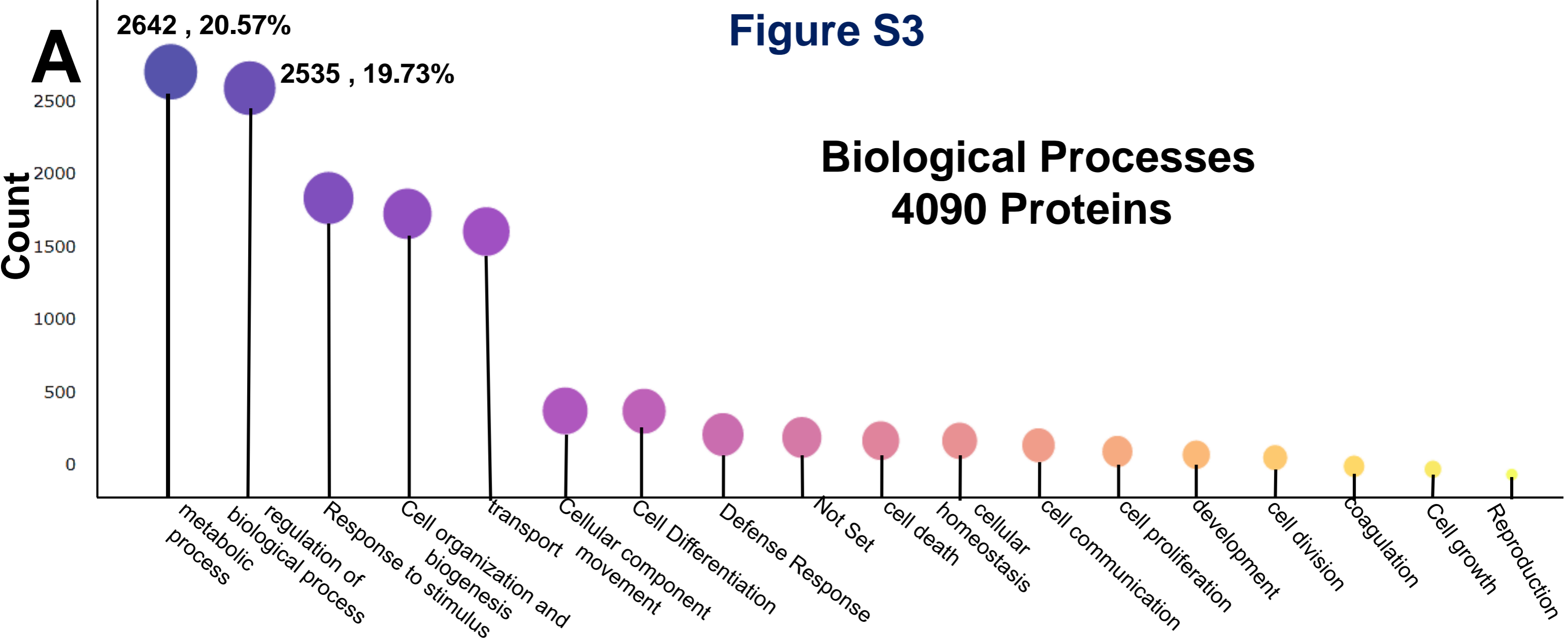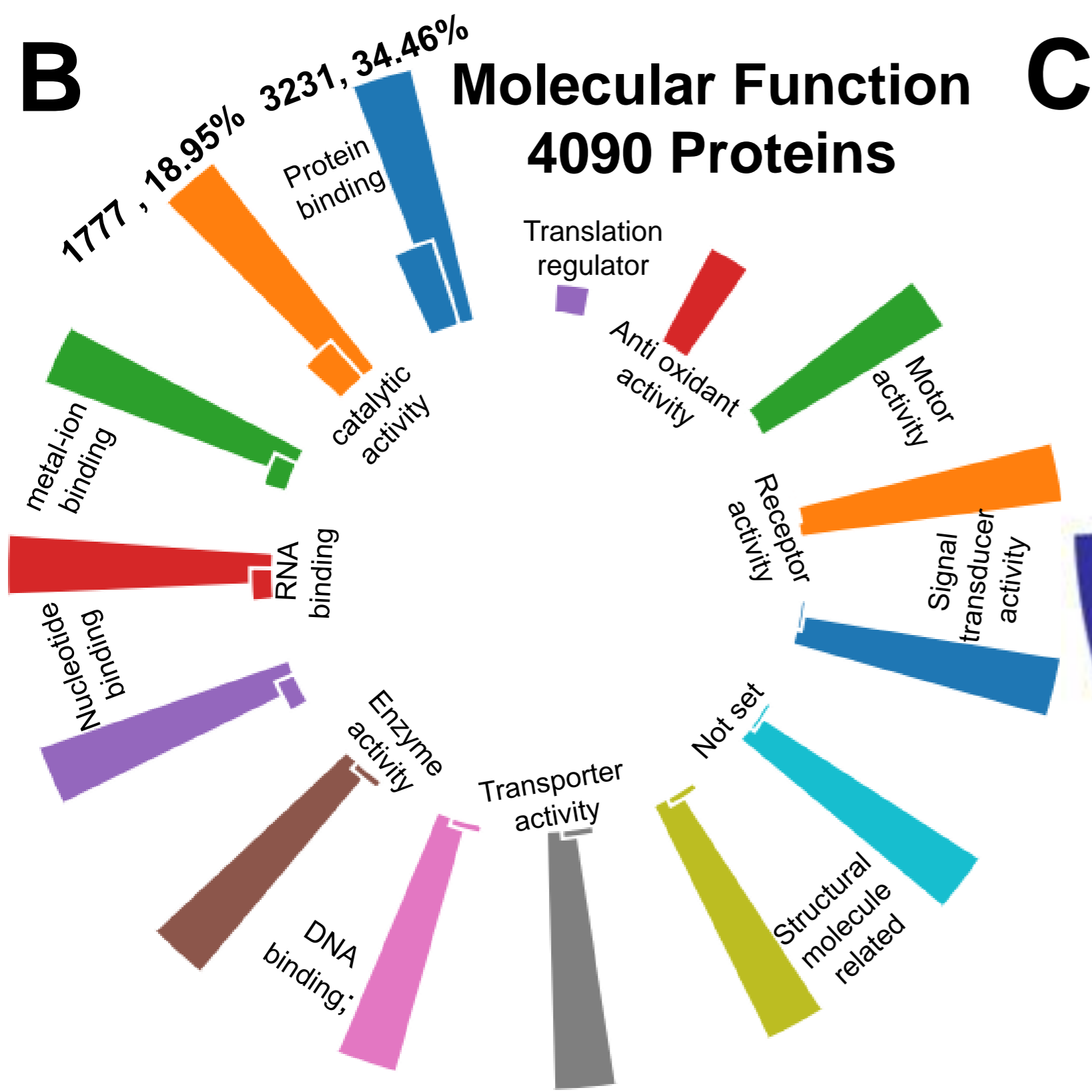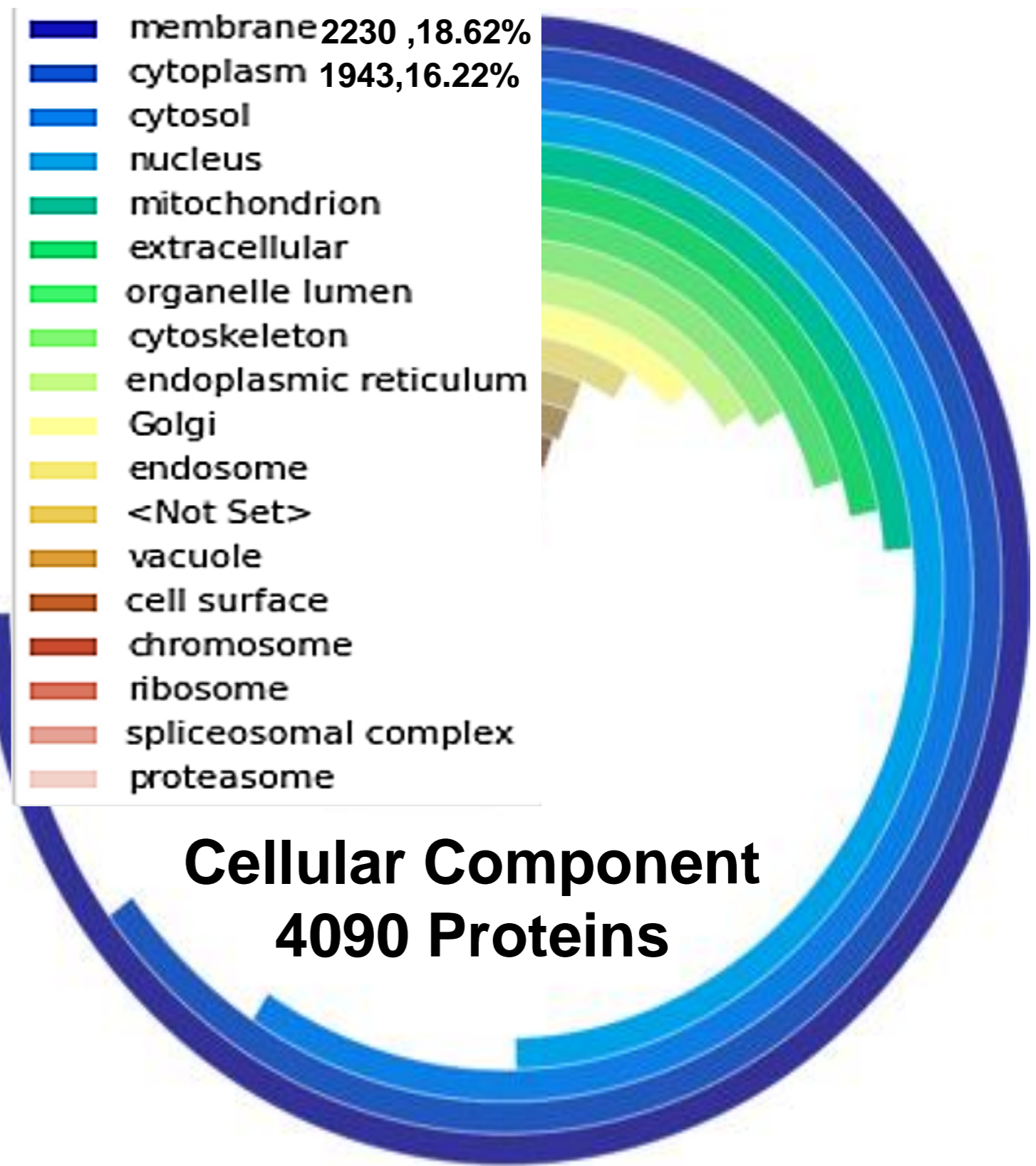

Supplement: Figure S3 [file mmc3.pdf]

Figure S4

A

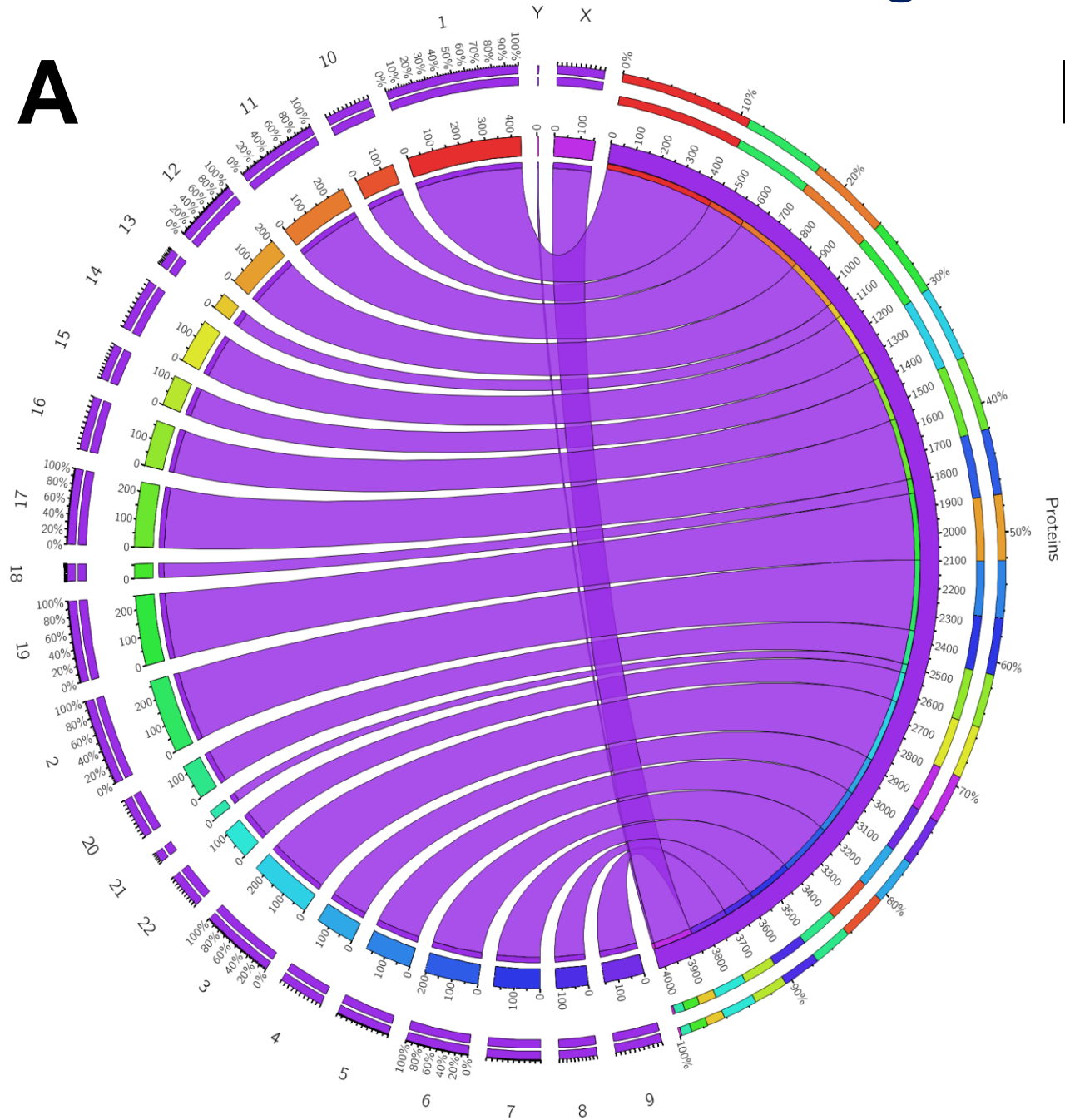

B

## Chromosomal Map of Pituitary Proteome

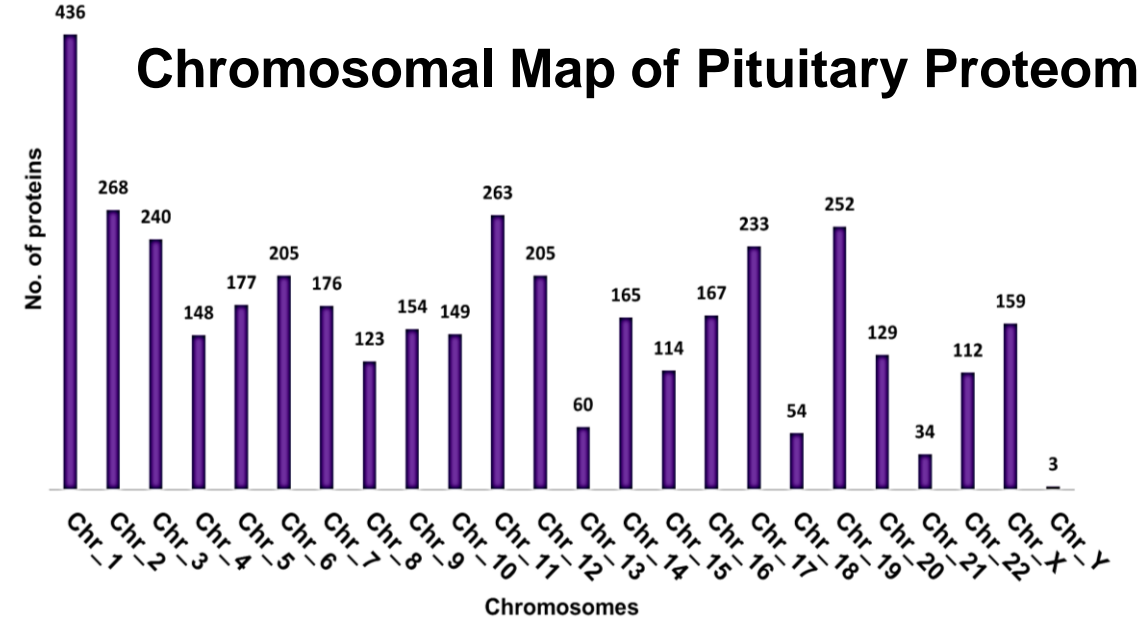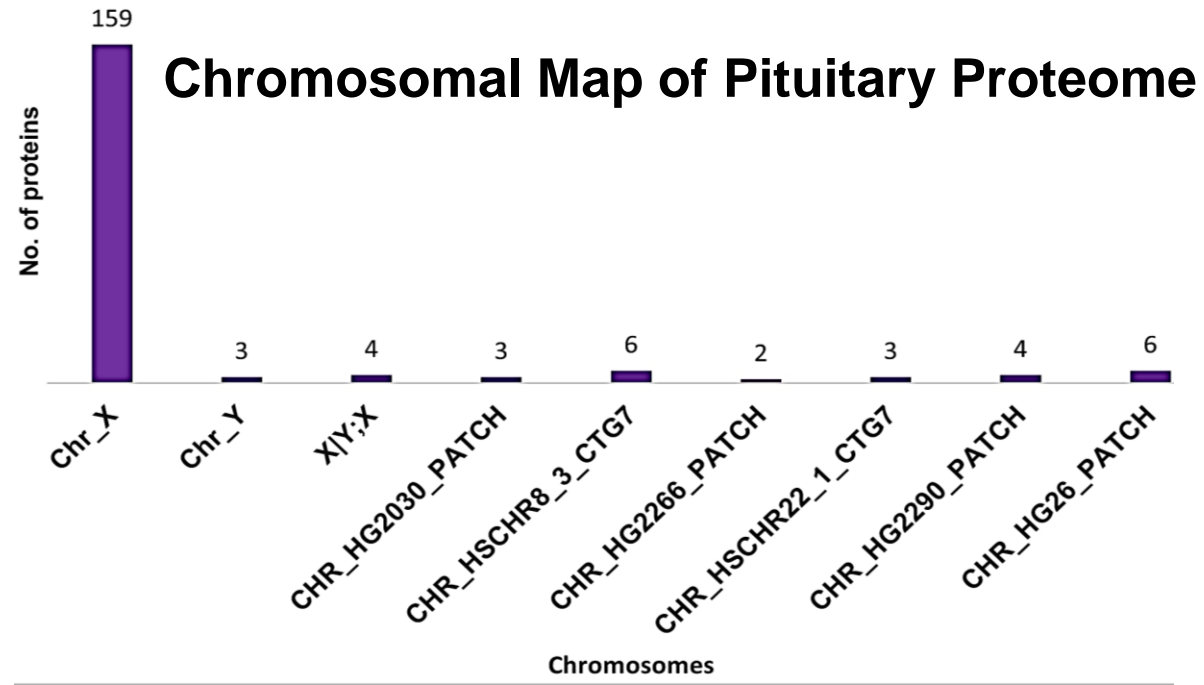

Supplement: Figure S4 [file mmc4.pdf]
