# Supplementary material for: The First Pituitary Proteome Landscape From Matched Anterior and Posterior Lobes for a Better Understanding of the Pituitary Gland
Source: Mol Cell Proteomics. 2022 Dec 5;22(1):100478. doi: 10.1016/j.mcpro.2022.100478 (PMC9877467; doi:10.1016/j.mcpro.2022.100478)
Supplement: Figure S2 [file mmc2.pdf]

Figure S2

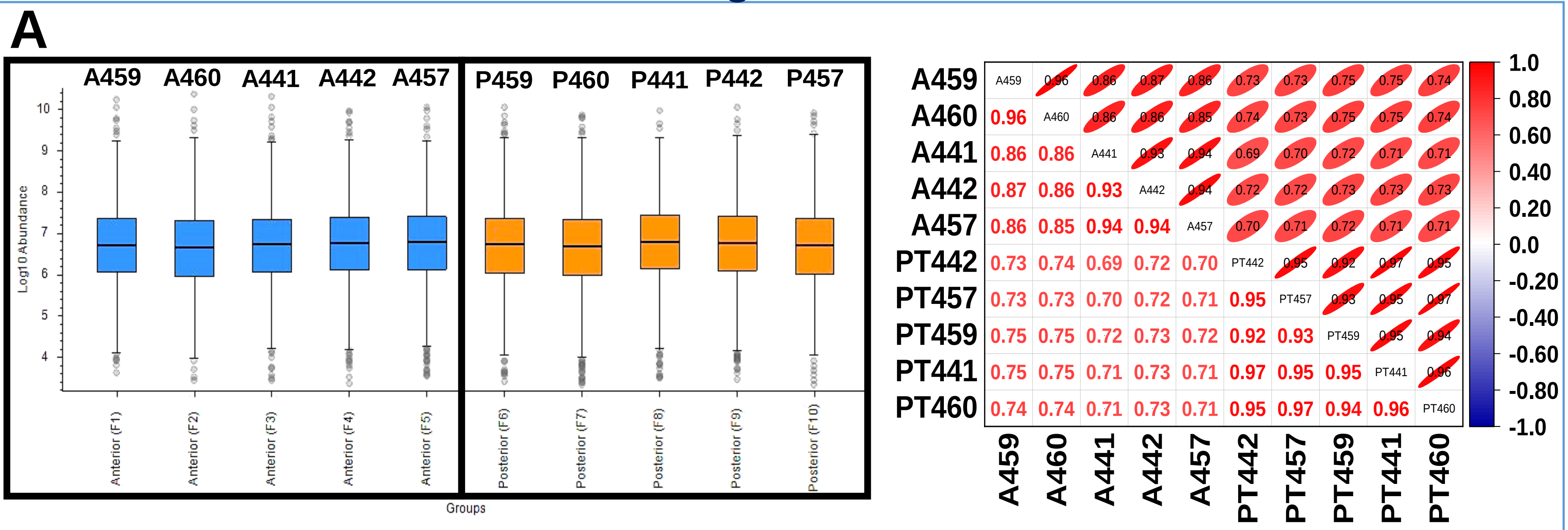

Log10 Normalised Abundance plot of each group and Pearson Correlation Plot

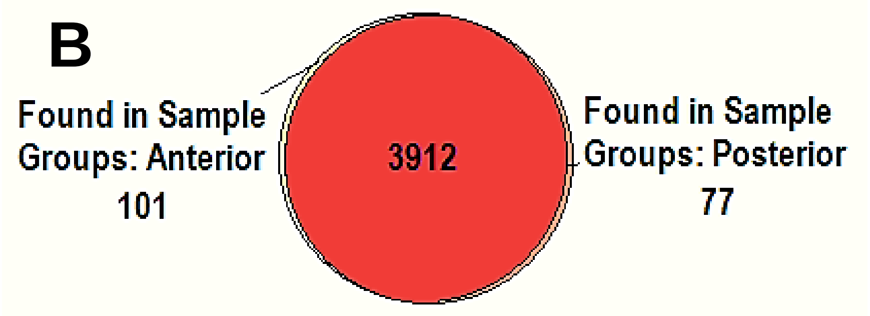

	Exclusive	Total	Label
A	101	4013	Found in Sample Groups : Anterior
B	77	3989	Found in Sample Groups : Posterior
A|B	3912	3912	Found in Sample Groups : (Anterior | Posterior)
Sum	4090		

Venn Diagram of Proteins found and Anterior and Posterior Pituitary Lobes

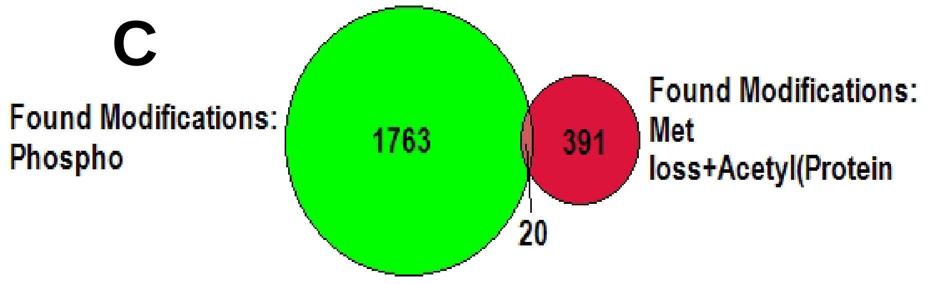

	Exclusive	Total	Label
A	1763	1783	Found Modification: Phospho
B	391	411	Found Modification: Met-Loss+ Acetyl(Protein N-Term)
A|B	20	20	Found Modification: Phospho|Met-Loss+ Acetyl(Protein N-Term)
Sum	2174		

Venn Diagram of Peptide Groups found modifications: Phospho and Met-Loss+ Acetyl(Protein N-Term)

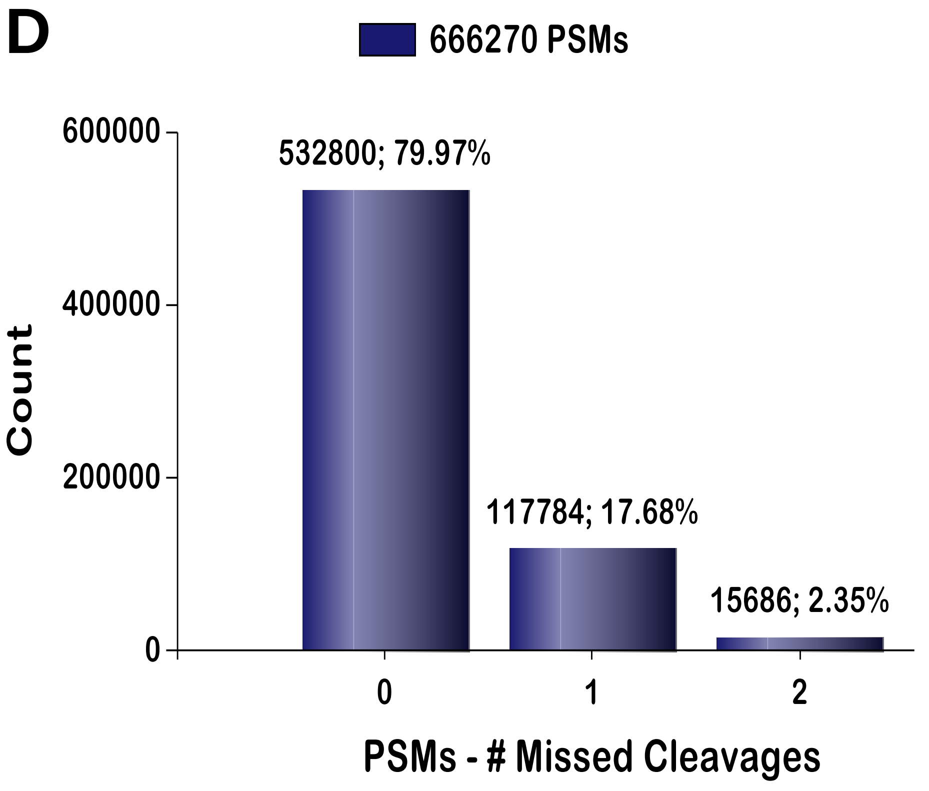

Bar Chart of PSMs # Missed Cleavages

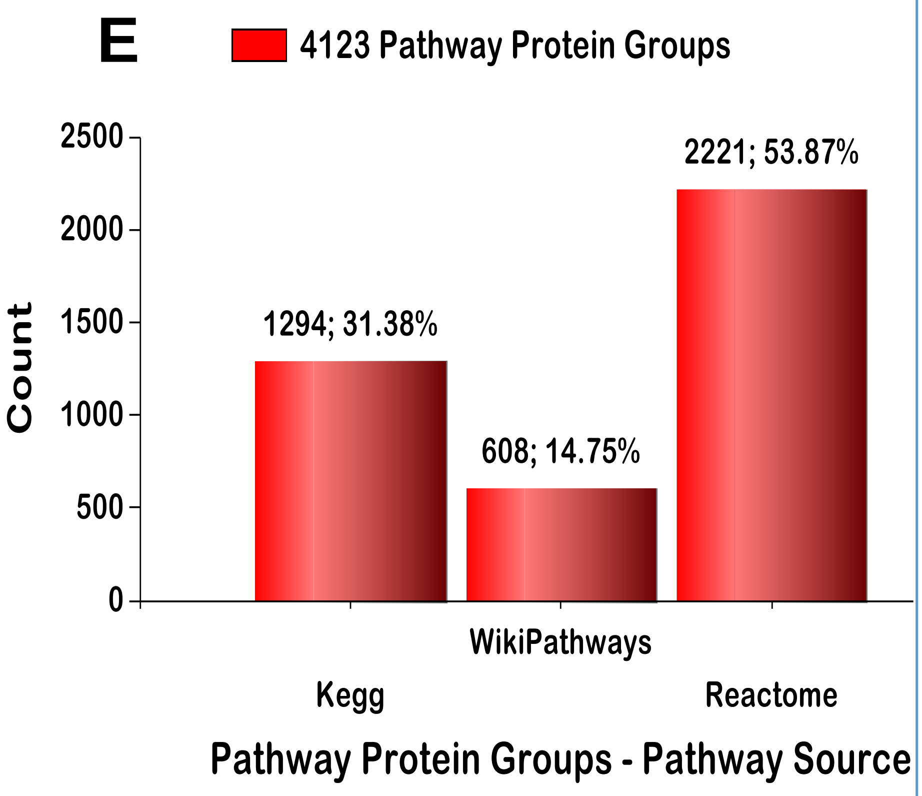

Bar Chart of Pathway Protein group source
