# Supplementary material for: The First Pituitary Proteome Landscape From Matched Anterior and Posterior Lobes for a Better Understanding of the Pituitary Gland
Source: Mol Cell Proteomics. 2022 Dec 5;22(1):100478. doi: 10.1016/j.mcpro.2022.100478 (PMC9877467; doi:10.1016/j.mcpro.2022.100478)
Supplement: Figure S5 [file mmc5.pdf]

Figure S5

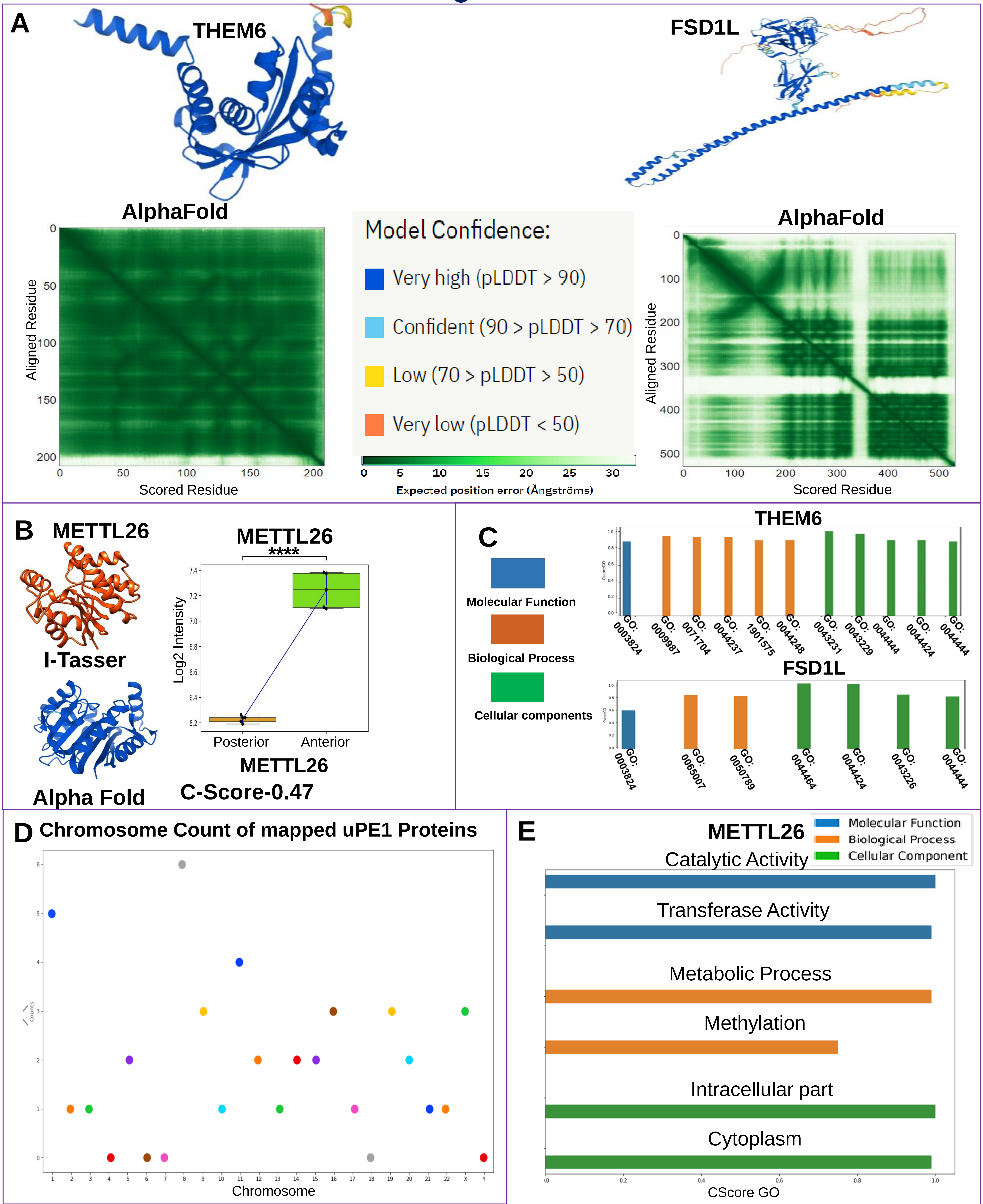
A
THEM6
FSD1L
AlphaFold
Aligned Residue
Scored Residue
Model Confidence:
Very high (pLDDT > 90)
Confident (90 > pLDDT > 70)
Low (70 > pLDDT > 50)
Very low (pLDDT < 50)
Expected position error (Ångströms)
AlphaFold
Aligned Residue
Scored Residue
B
METTL26
I-Tasser
Alpha Fold
METTL26
****
Log2 Intensity
Posterior
Anterior
METTL26
C-Score-0.47
C
Molecular Function
Biological Process
Cellular components
THEM6
GO: 0003824
GO: 0009987
GO: 0071704
GO: 0044237
GO: 1901575
GO: 0044248
GO: 0043231
GO: 0043229
GO: 0044444
GO: 0044424
GO: 0044444
FSD1L
GO: 0003824
GO: 0065007
GO: 0050789
GO: 0044464
GO: 0044424
GO: 0043226
GO: 0044444
D
Chromosome Count of mapped uPE1 Proteins
Counts
Chromosome
E
METTL26
Molecular Function
Biological Process
Cellular Component
Catalytic Activity
Transferase Activity
Metabolic Process
Methylation
Intracellular part
Cytoplasm
CScore GO
