# Supplementary material for: The First Pituitary Proteome Landscape From Matched Anterior and Posterior Lobes for a Better Understanding of the Pituitary Gland
Source: Mol Cell Proteomics. 2022 Dec 5;22(1):100478. doi: 10.1016/j.mcpro.2022.100478 (PMC9877467; doi:10.1016/j.mcpro.2022.100478)

Figure S6

A. String Analysis of Proteins significantly expressed in Anterior lobe

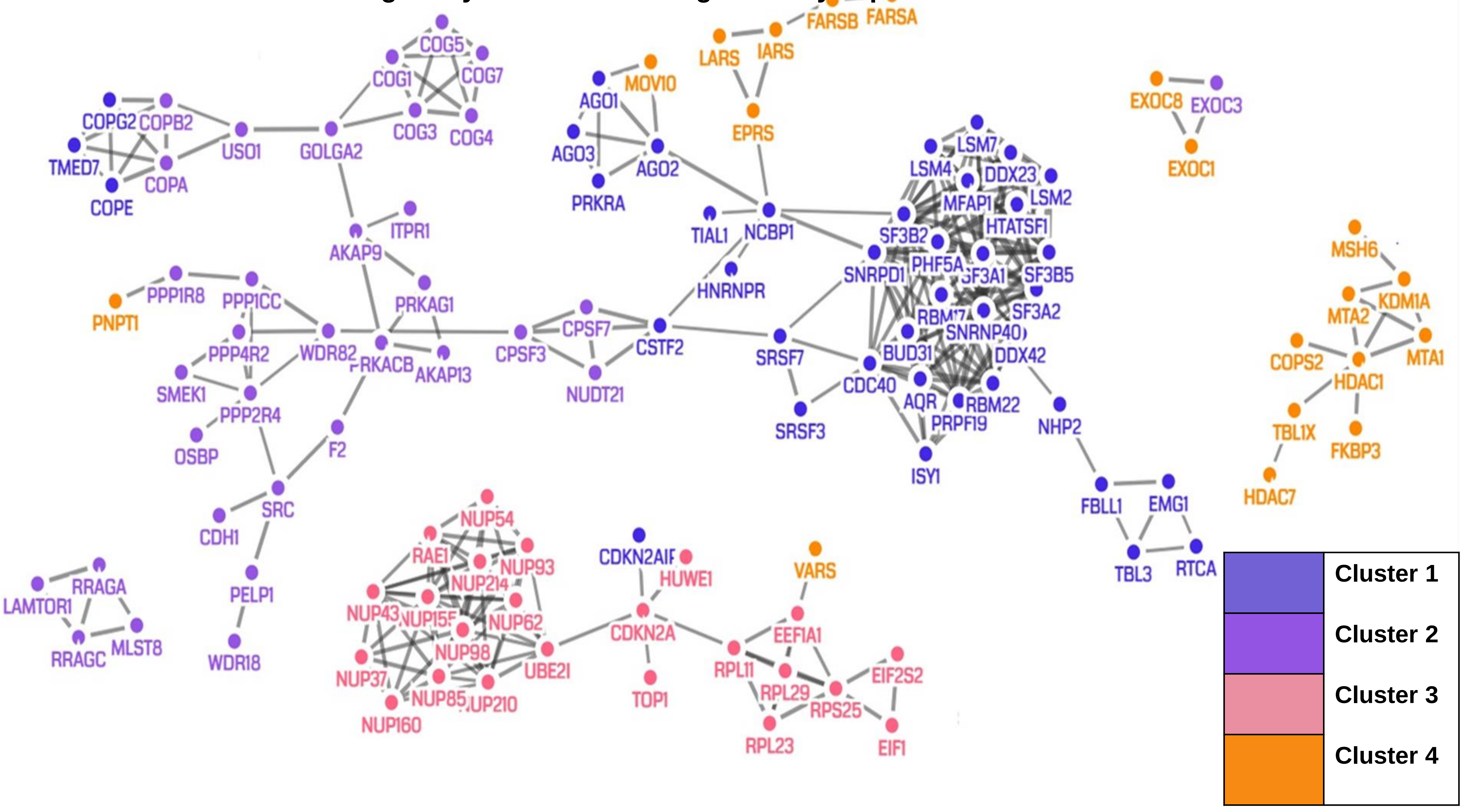

B. String Analysis of Proteins significantly expressed Posterior lobe

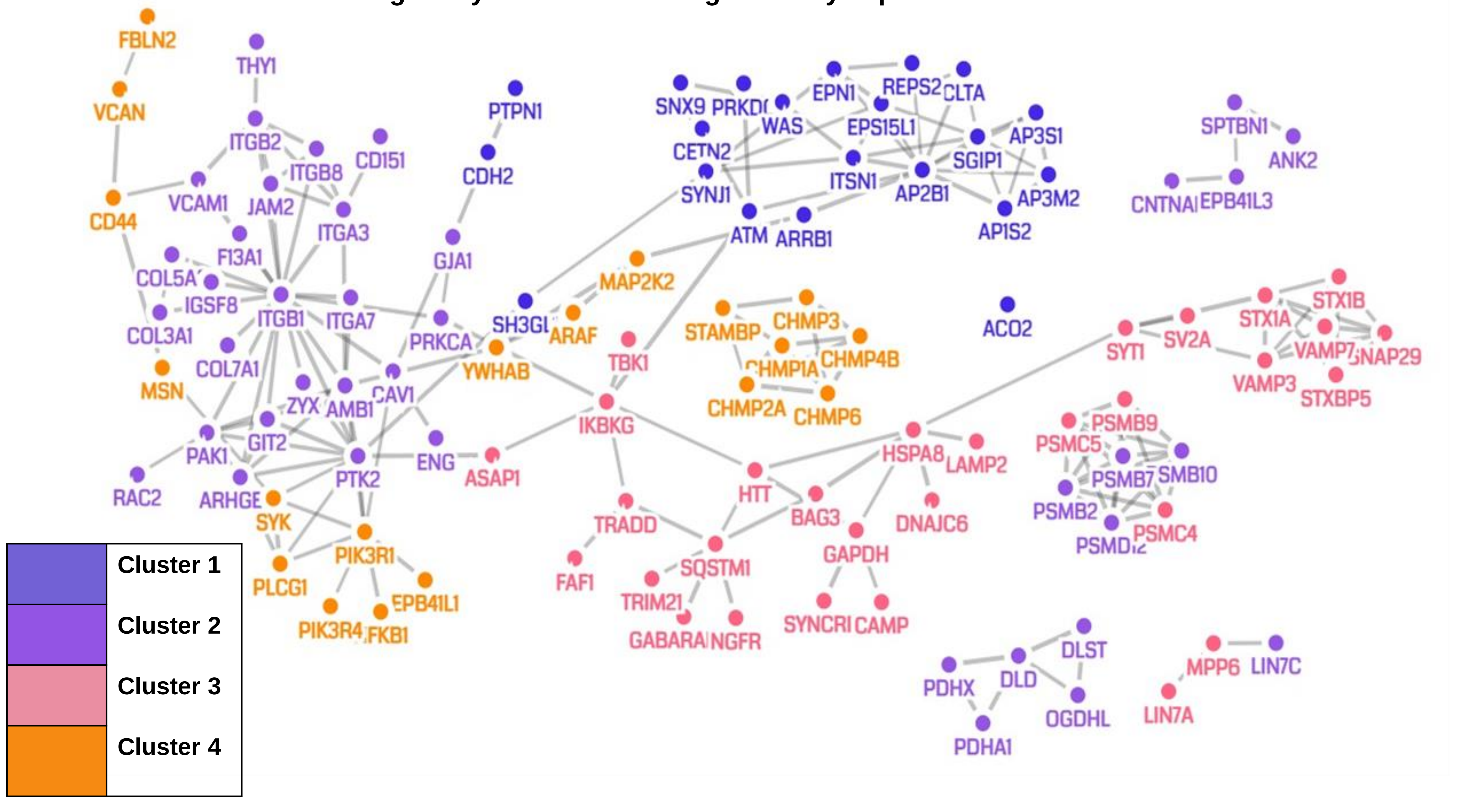

Supplement: Figure S6 [file mmc6.pdf]
