# Supplementary material for: The First Pituitary Proteome Landscape From Matched Anterior and Posterior Lobes for a Better Understanding of the Pituitary Gland
Source: Mol Cell Proteomics. 2022 Dec 5;22(1):100478. doi: 10.1016/j.mcpro.2022.100478 (PMC9877467; doi:10.1016/j.mcpro.2022.100478)

Figure S7

sp|P62805|H4_HUMAN R.ISGLIYEETR.G [46, 55]

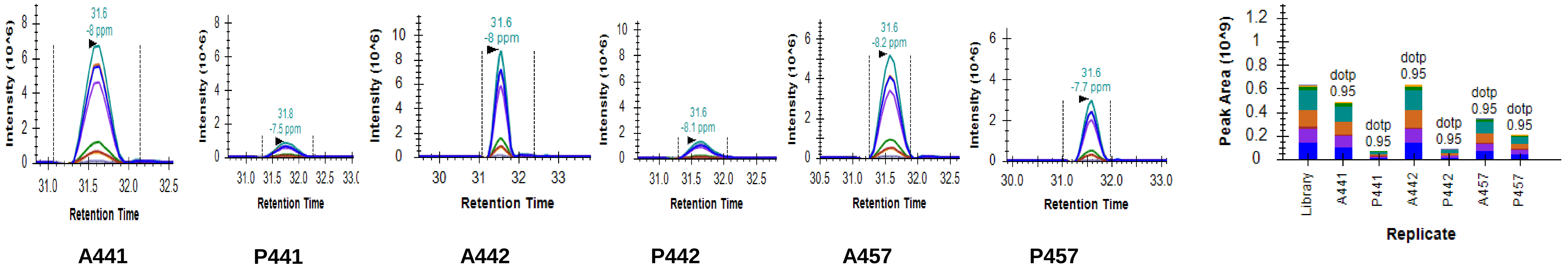

sp|P62805|H4_HUMAN

K.VFLENVIR.D [60, 67]

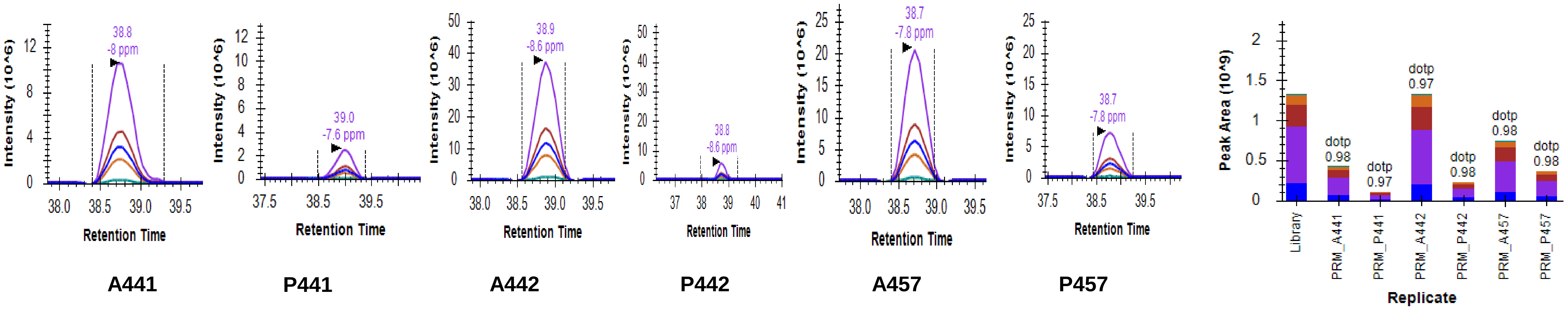

sp|P01241|SOMA_HUMAN

R.LHQLAFDTYQEFEEAYIPK.E [45, 63]

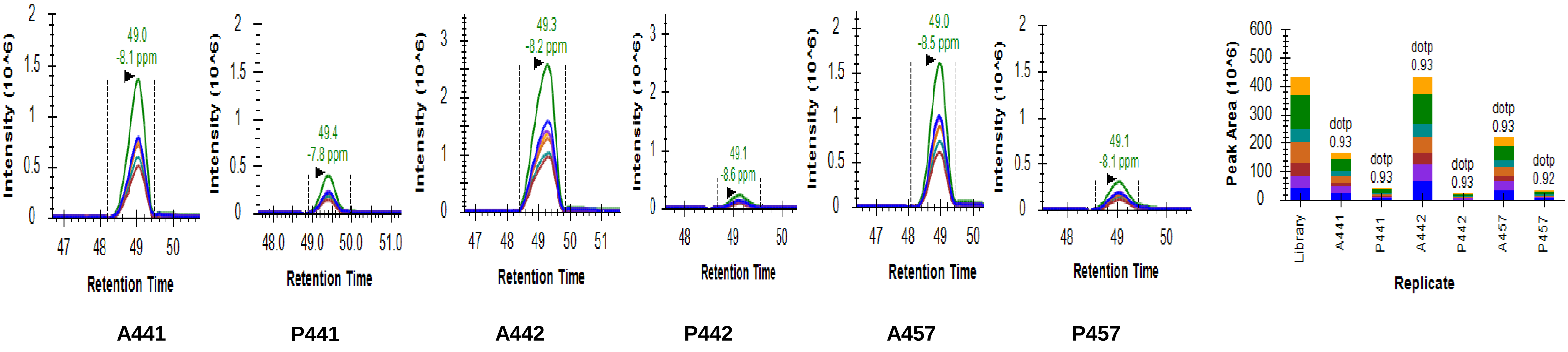

sp|P01241|SOMA_HUMAN

R.ISLLLIQSWLEPVQFLR.S [103, 119]

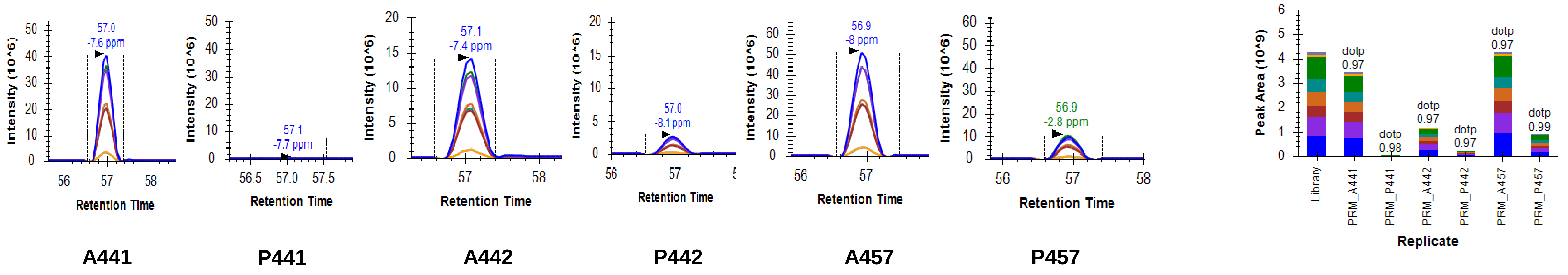

sp|P01241|SOMA_HUMAN

K.FDTNSHNDDALLK.N [171, 183]

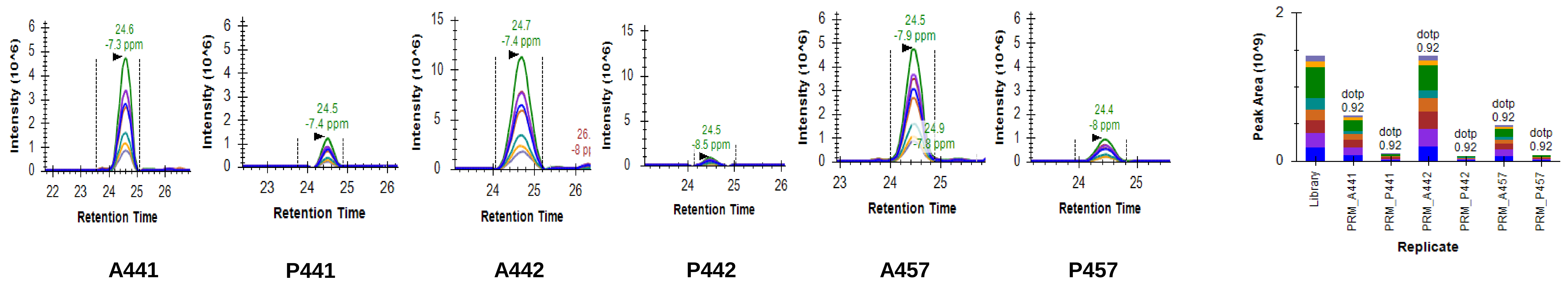

sp|Q9Y2W1|TR150_HUMAN

K.SPLQSVVVR.R [252, 260]

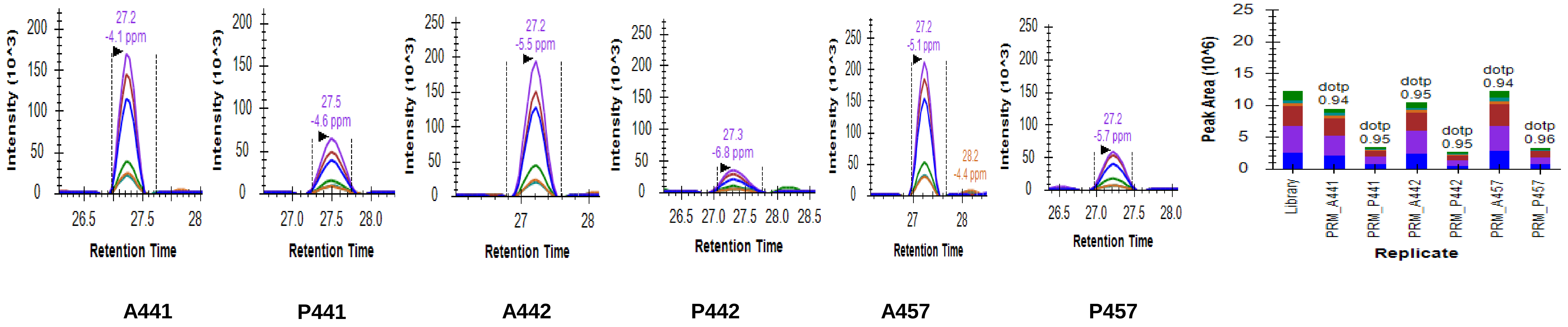

sp|Q9Y2W1|TR150_HUMAN

K.ESEFDDEPK.F [442, 450]

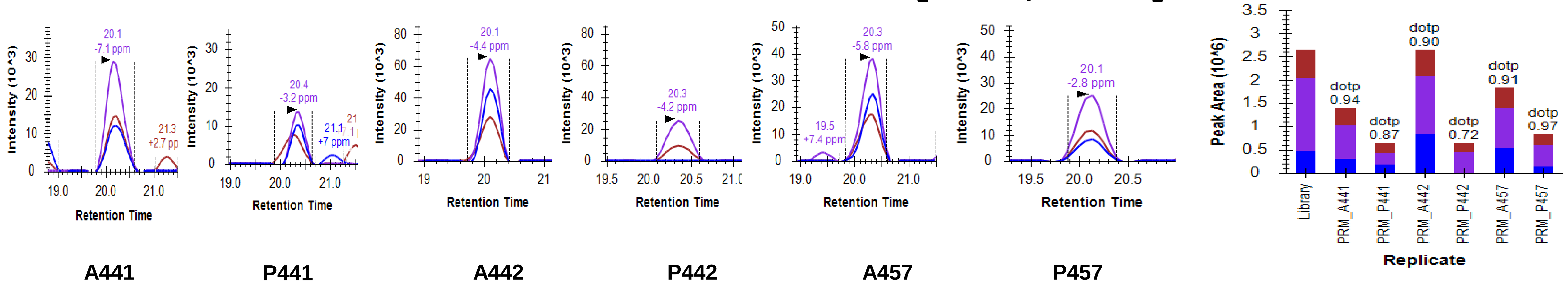

sp|P01222|TSHB_HUMAN

K.YALSQDVCTYR.D [64, 74]

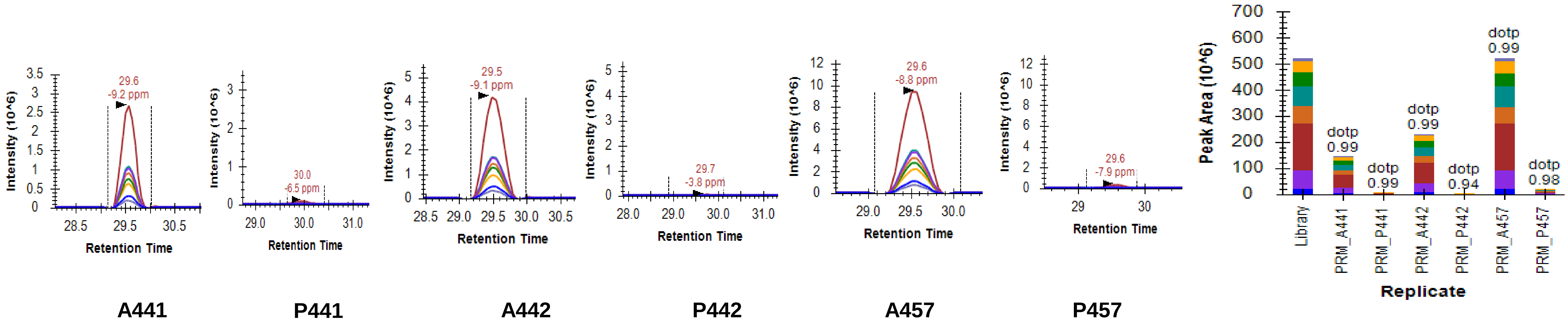

sp|P01222|TSHB_HUMAN

K.CNTDYSDCIHEAIK.T [107, 120]

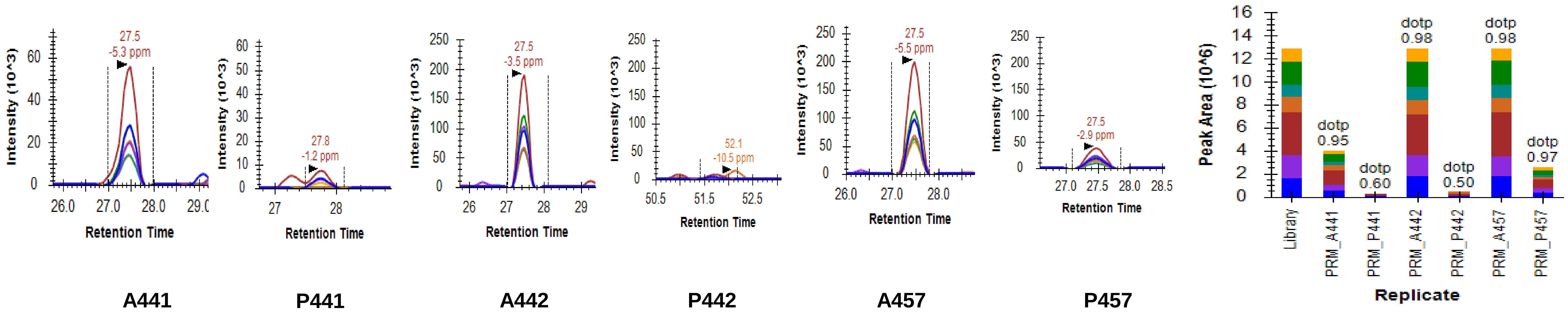

sp|P01185|NEU2_HUMAN

R.AMSDLELR.Q [31, 38]

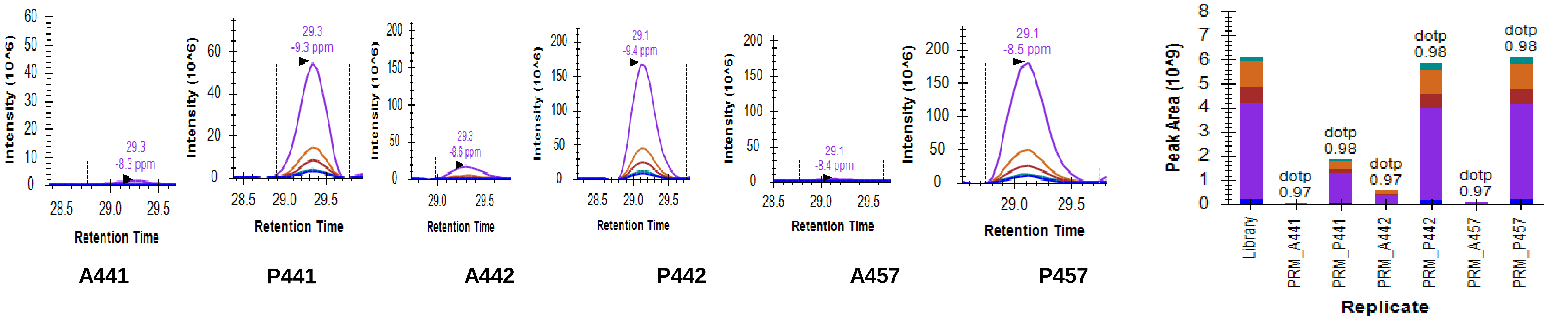

sp|P01185|NEU2_HUMAN

R.QCLPCGPGGK.G [39, 48]

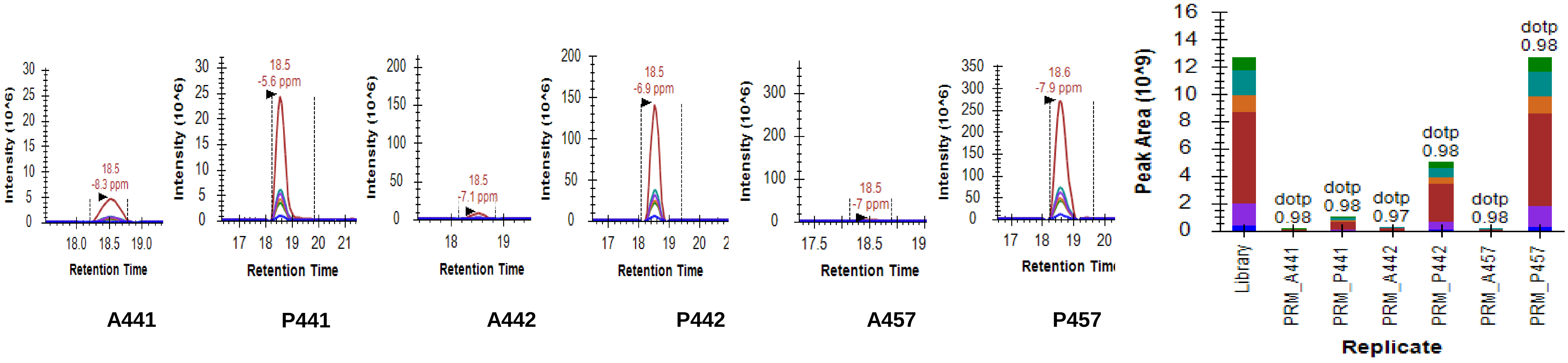

sp|P01185|NEU2_HUMAN

R.CAAFGVCCNDESCVTEPECR.E [97, 116]

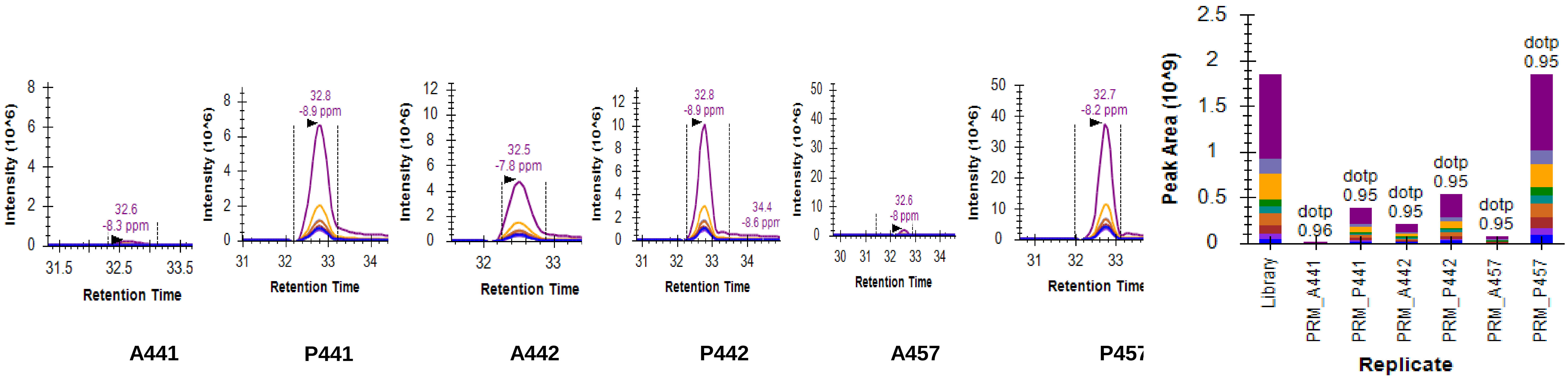

sp|P01178|NEU1_HUMAN

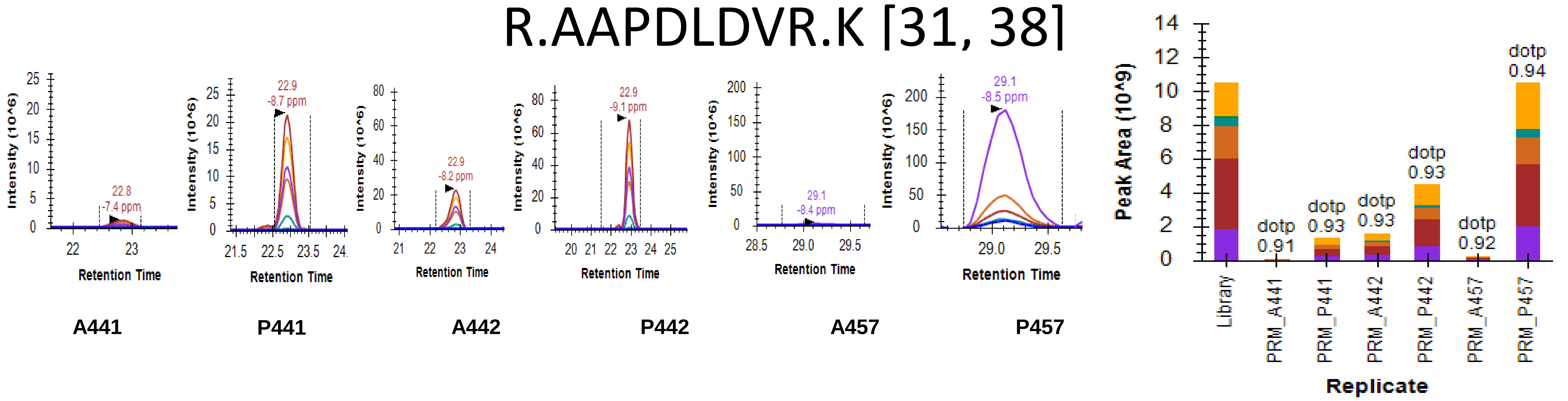

sp|P01185|NEU1_HUMAN

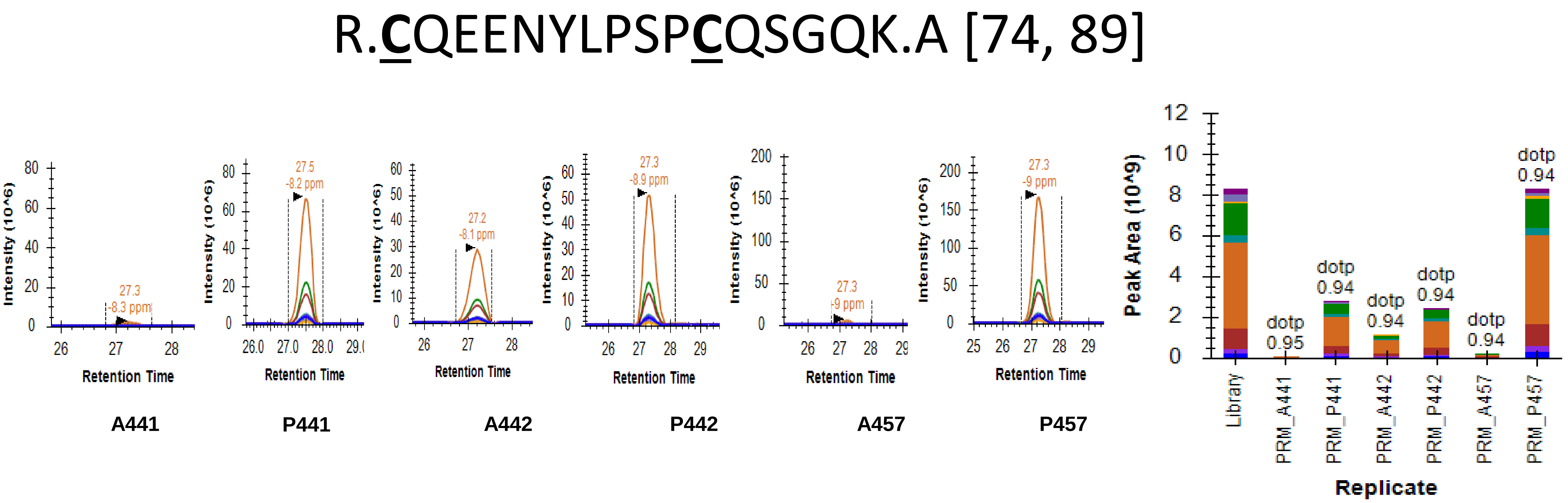

sp|P01189|COLI_HUMAN

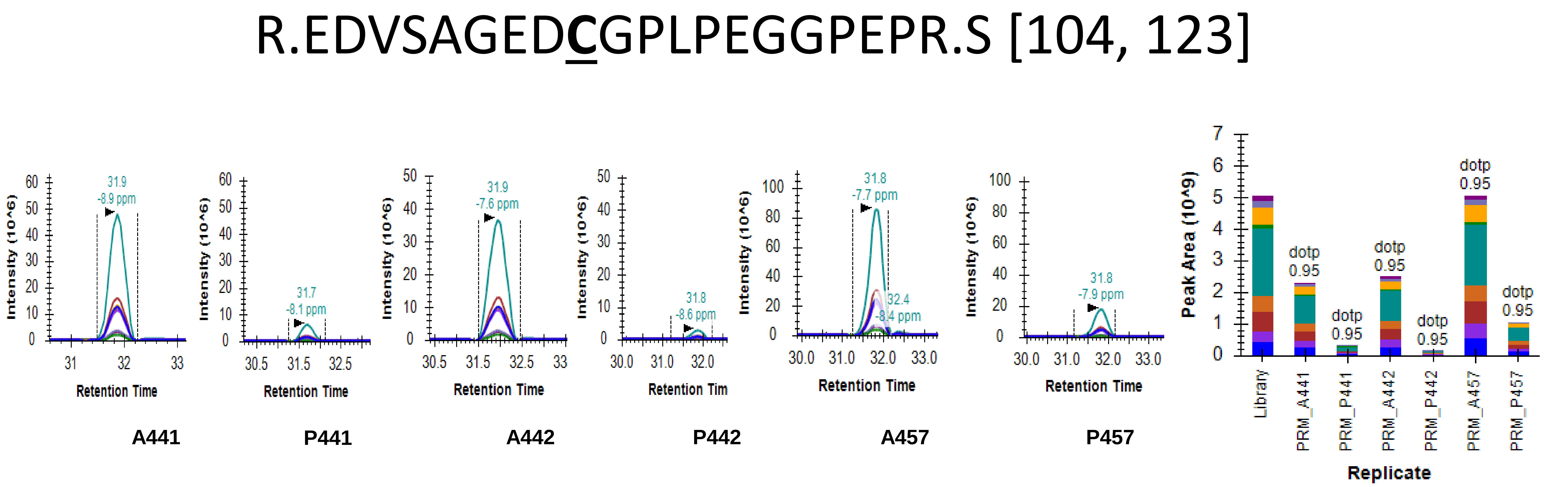

sp|P01189|COLI_HUMAN

R.SYSMEHFR.W [137, 144]

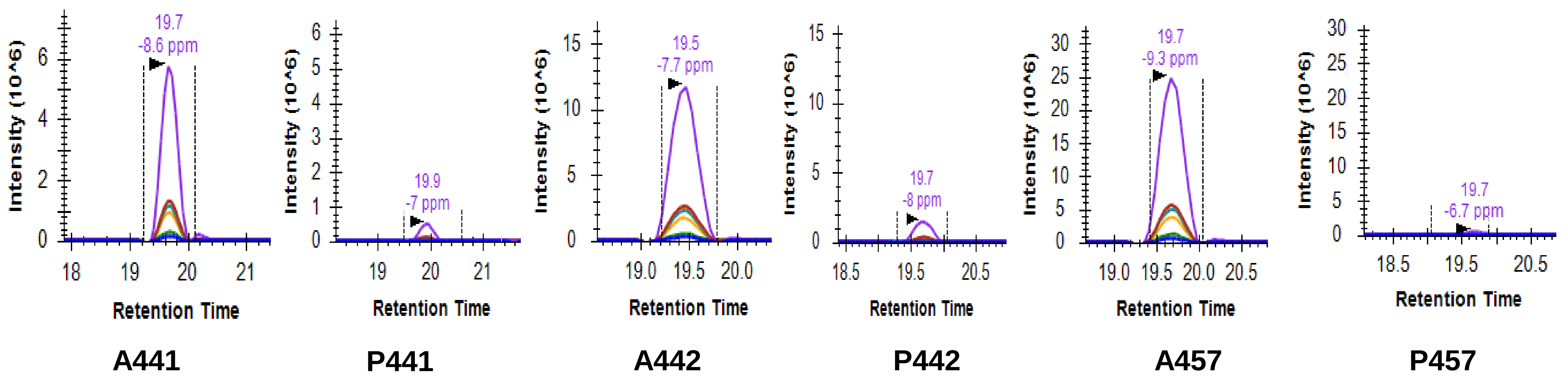

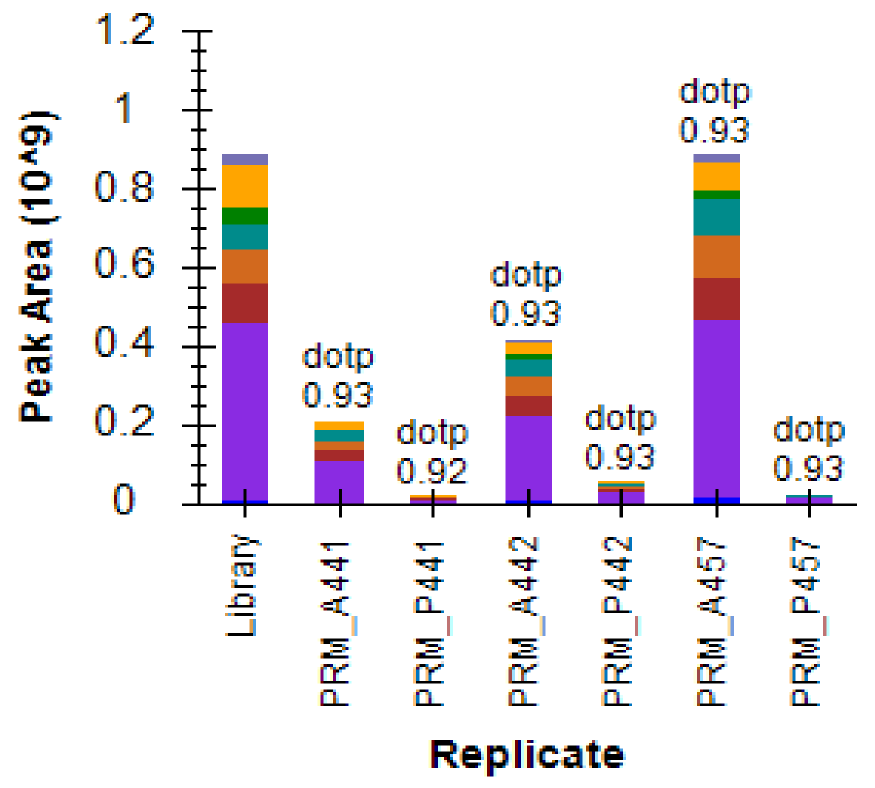

sp|P01189|COLI_HUMAN

K.VYPNGAEDESAEAFPLEFK.R [158, 176]

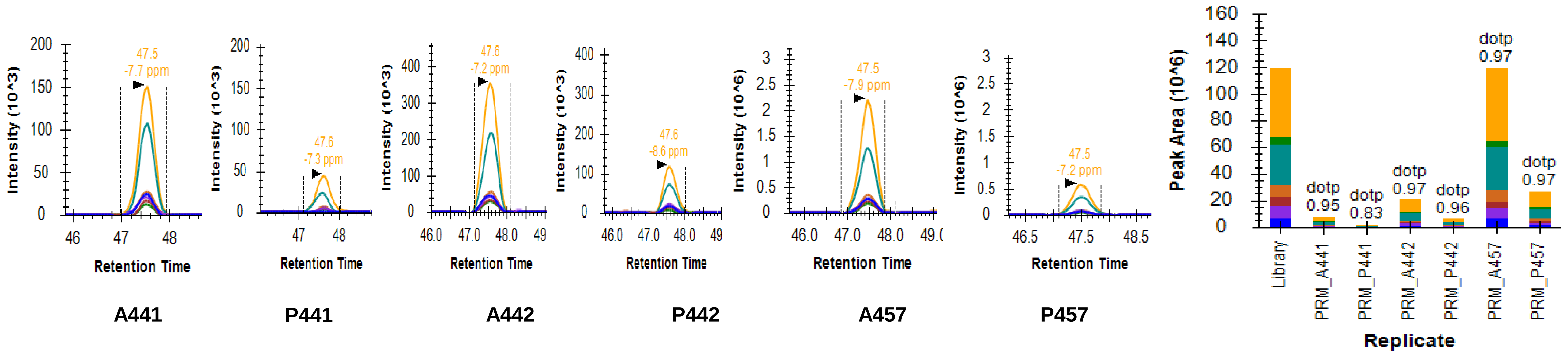

sp|P01189|COLI_HUMAN

K.SQTPLVTLFK.N [245, 254]

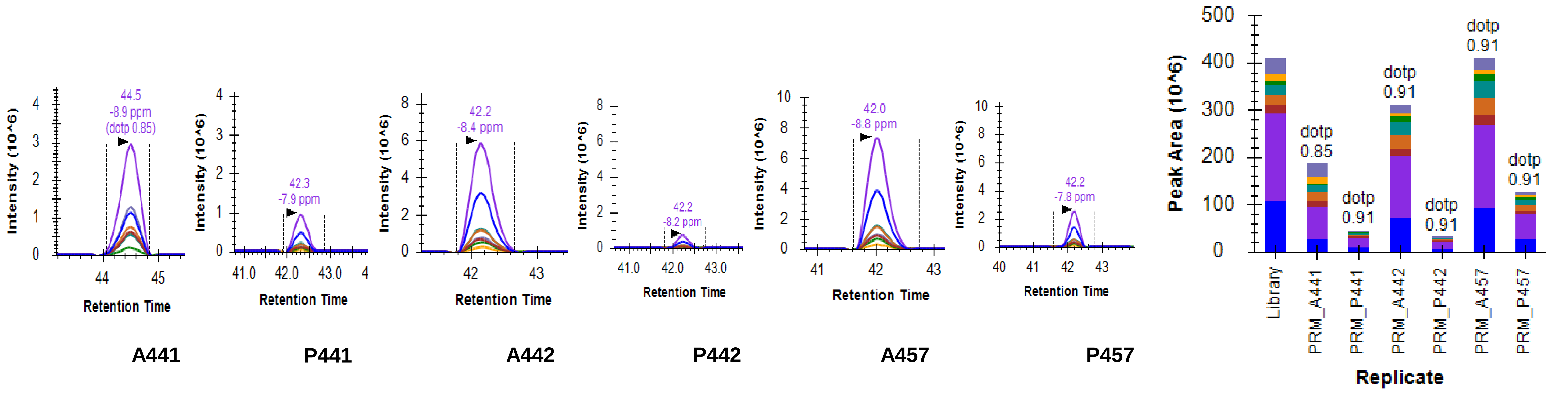

sp|Q14393|GAS6_HUMAN

R.SYSMEHFR.W [137, 144]

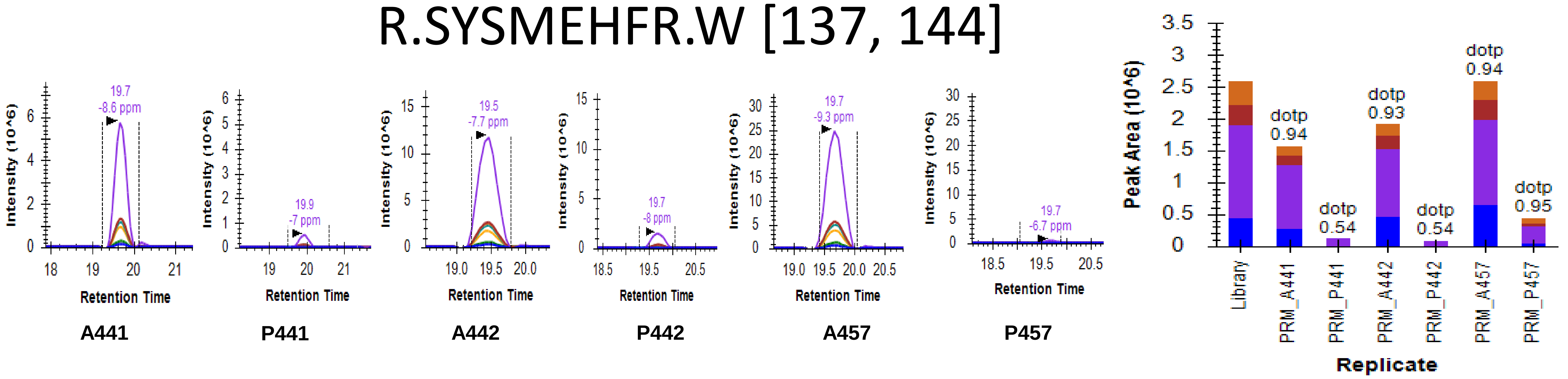

R.GQSEVSAAQLQER.L [594, 606]

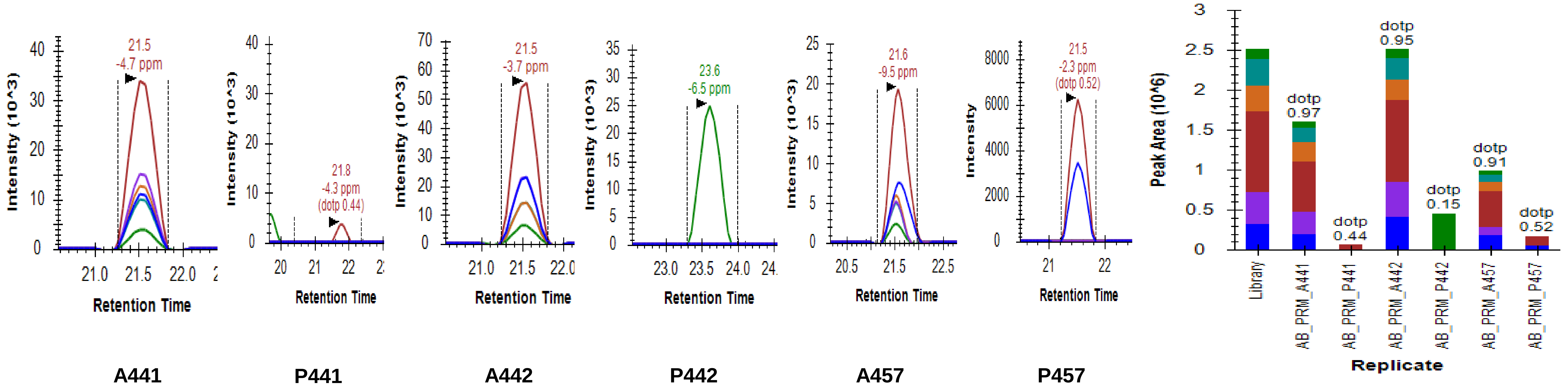

sp|Q96PC5|MIA2_HUMAN

K.DALNENSQLQESQK.Q [817, 830]

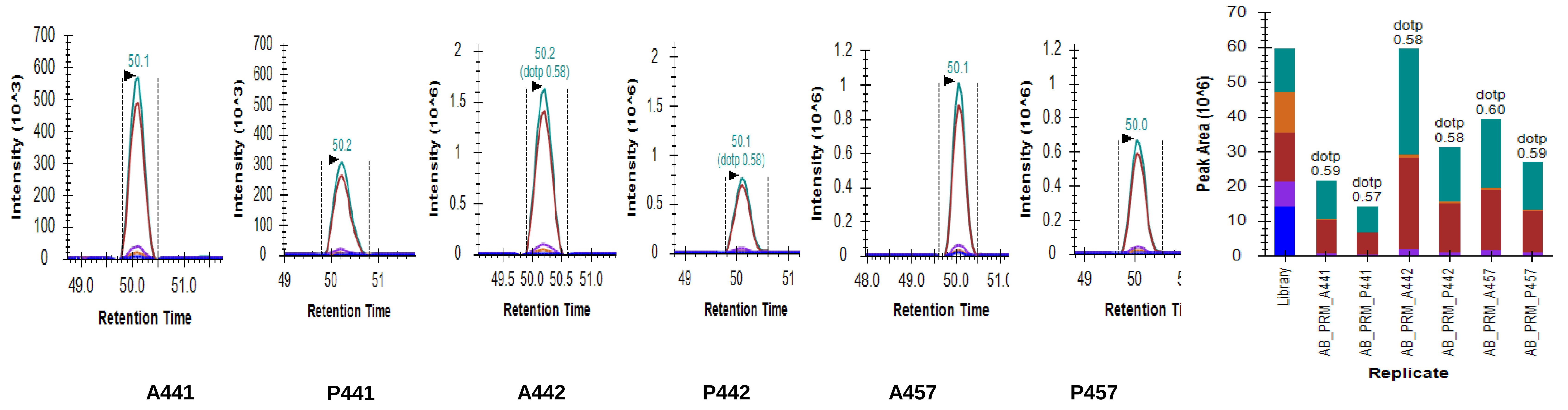

sp|P28069|PIT1_HUMAN

K.LVEEPIDMDSPEIR.E [117, 130]

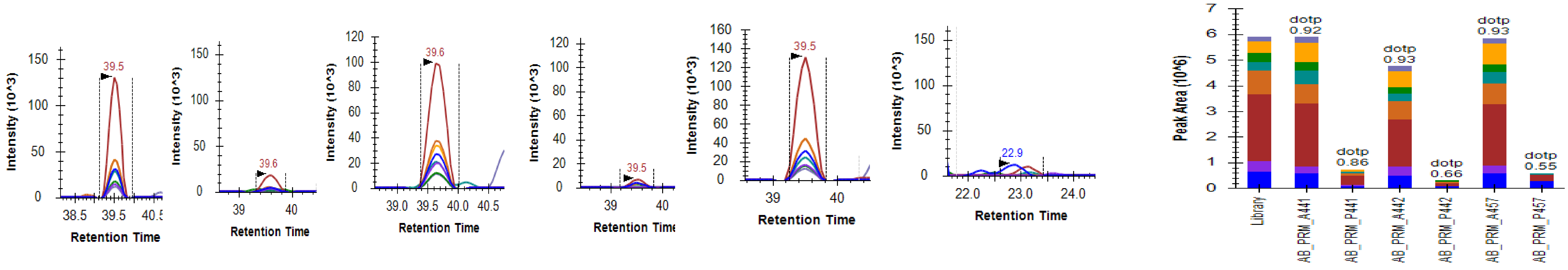

A441 P441 A442 P442 A457 P457

sp|P47972|NPTX2_HUMAN

K.DTMGDLPR.D [111, 118]

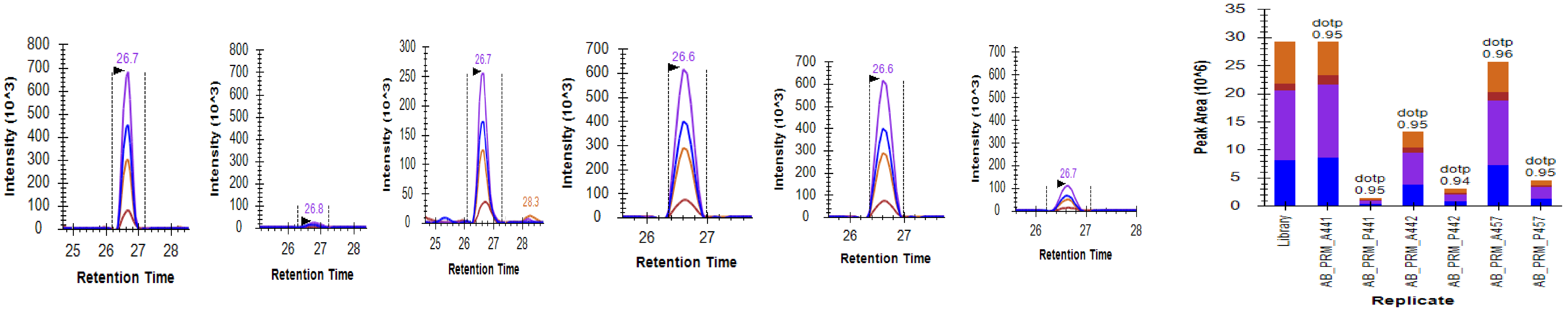

A441 P441 A442 P442 A457 P457

R.DPGHVVEQLSR.S [119, 129]

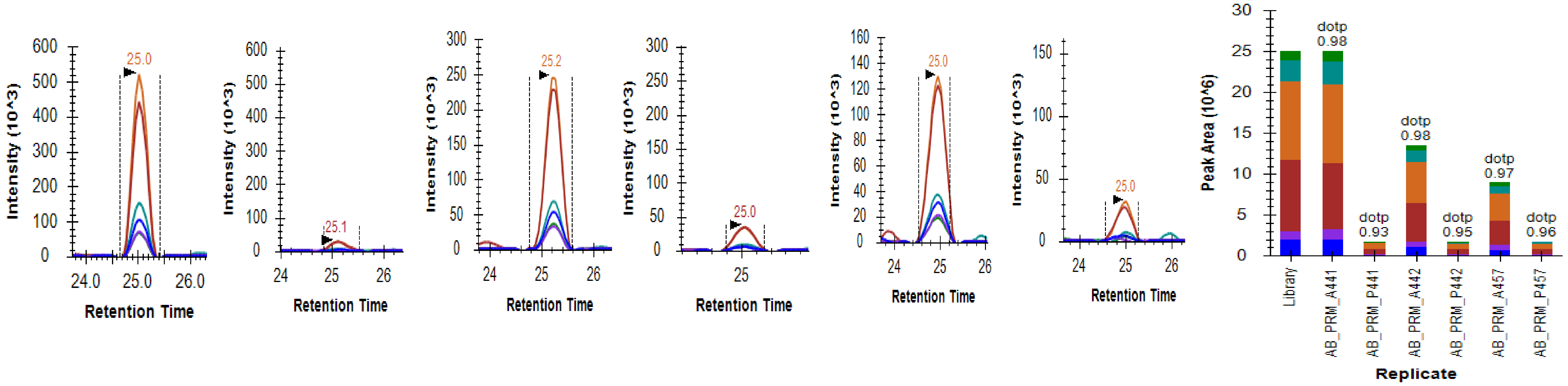

A441 P441 A442 P442 A457 P457

sp|Q9UKZ9|PCOC2_HUMAN

R.FIDLESDNL**C**R.Y [77, 87]

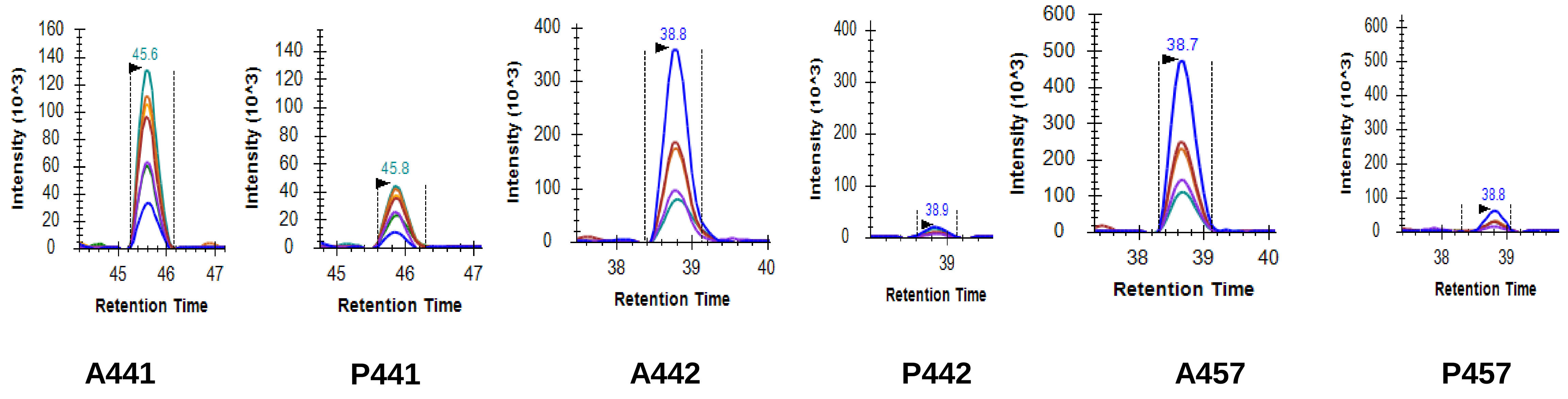

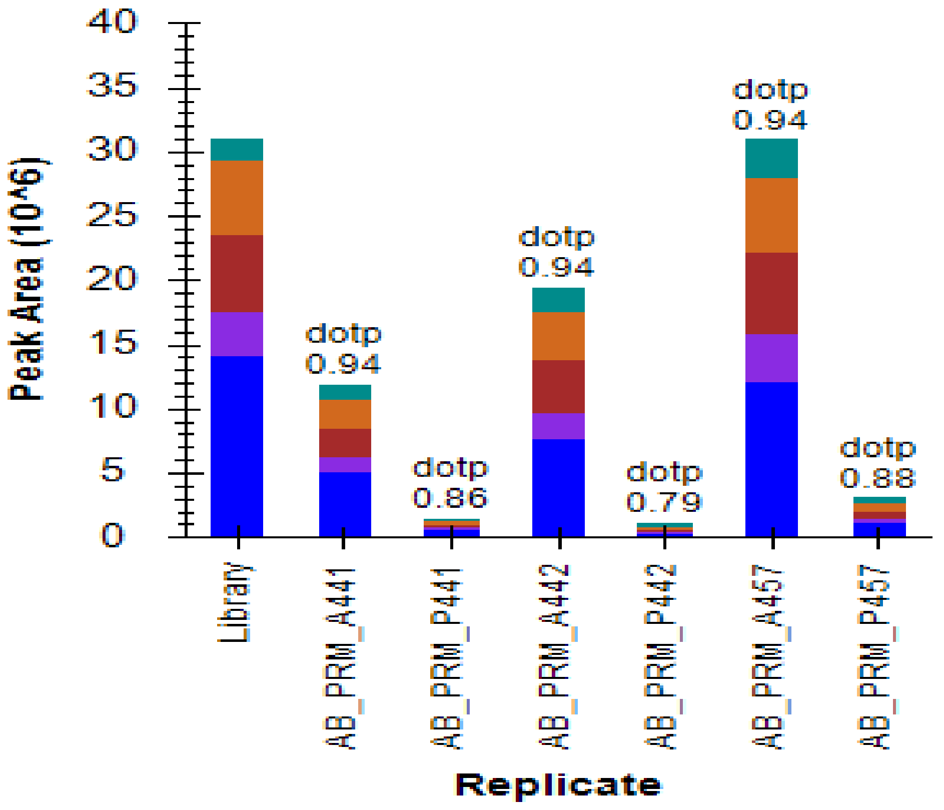

K.Y**C**GDSPPAPIVSER.N [230, 243]

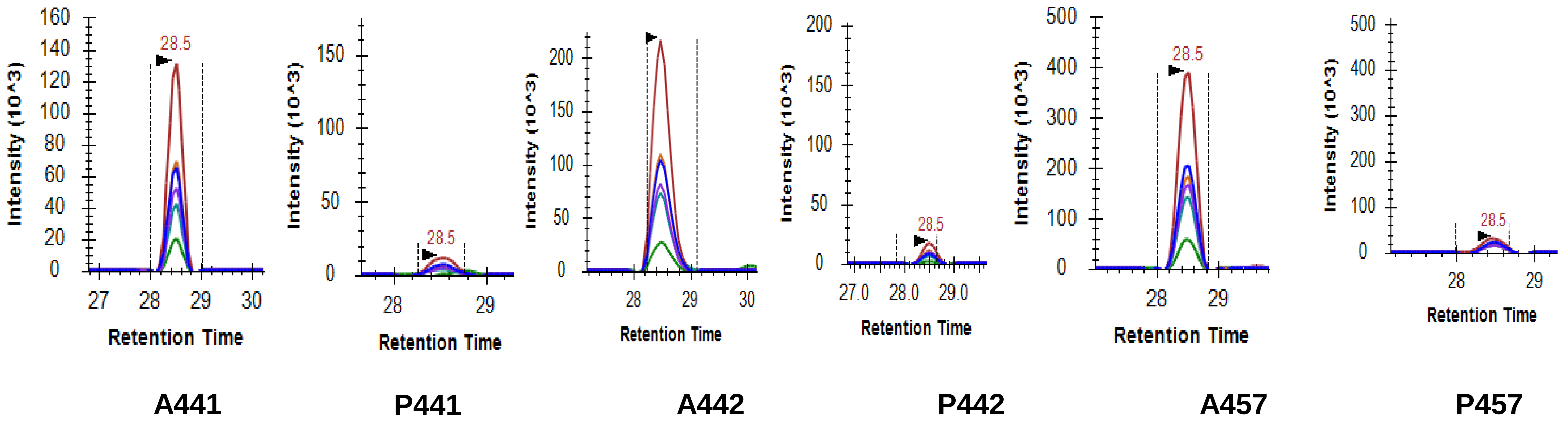

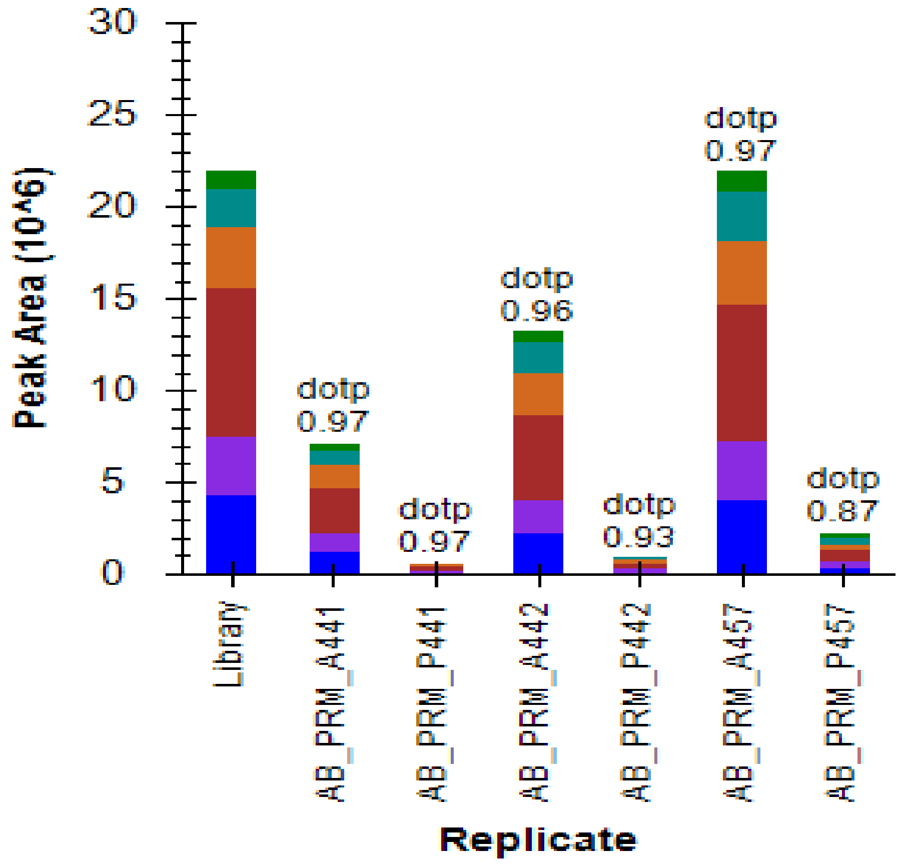

sp|O43719|HTSF1_HUMAN

K.LLDEDEIR.G [200, 207]

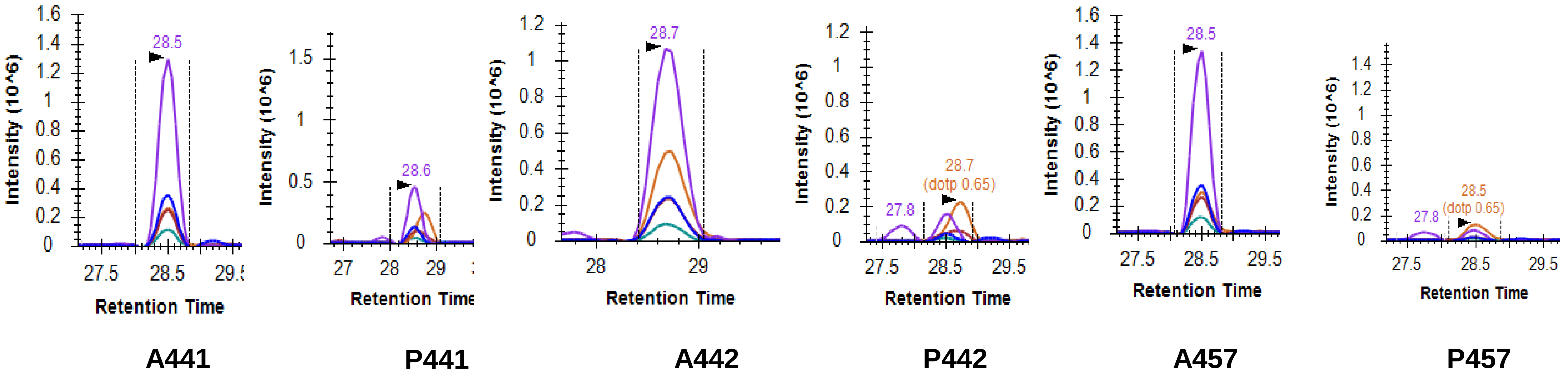

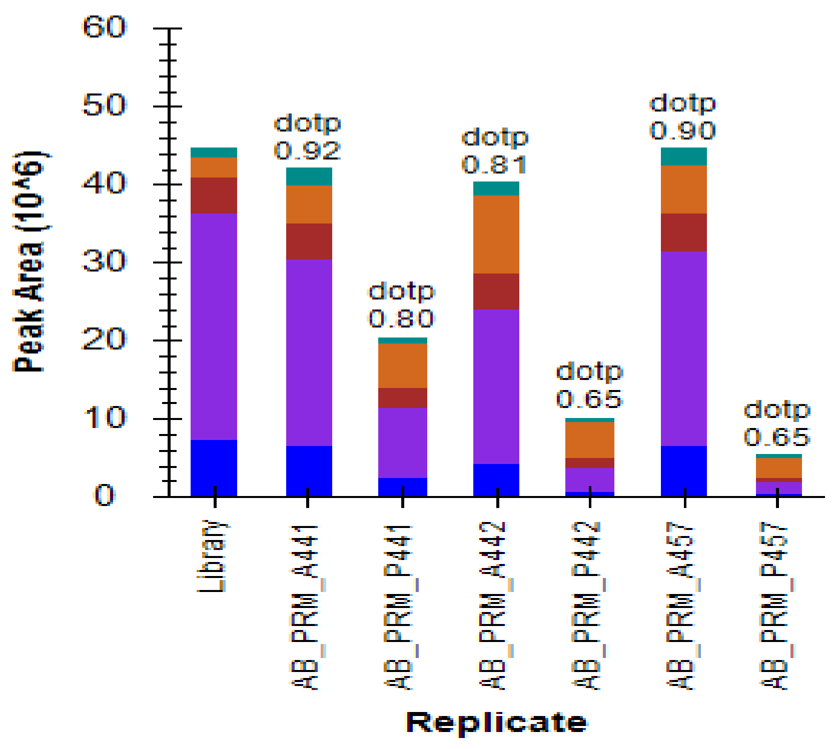

sp|O43719|HTSF1_HUMAN

R.GWEAFLNAPEANR.G [368, 380]

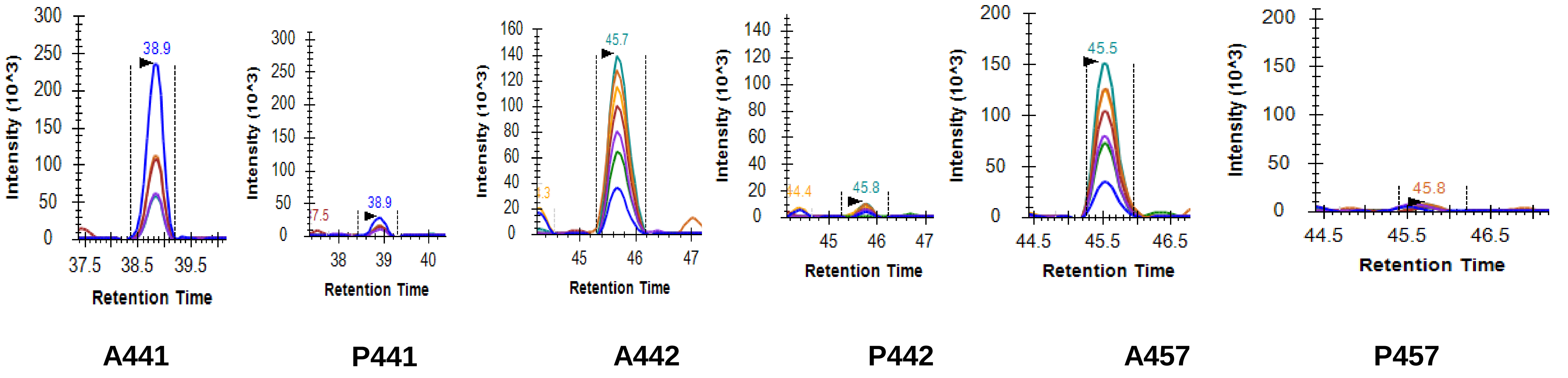

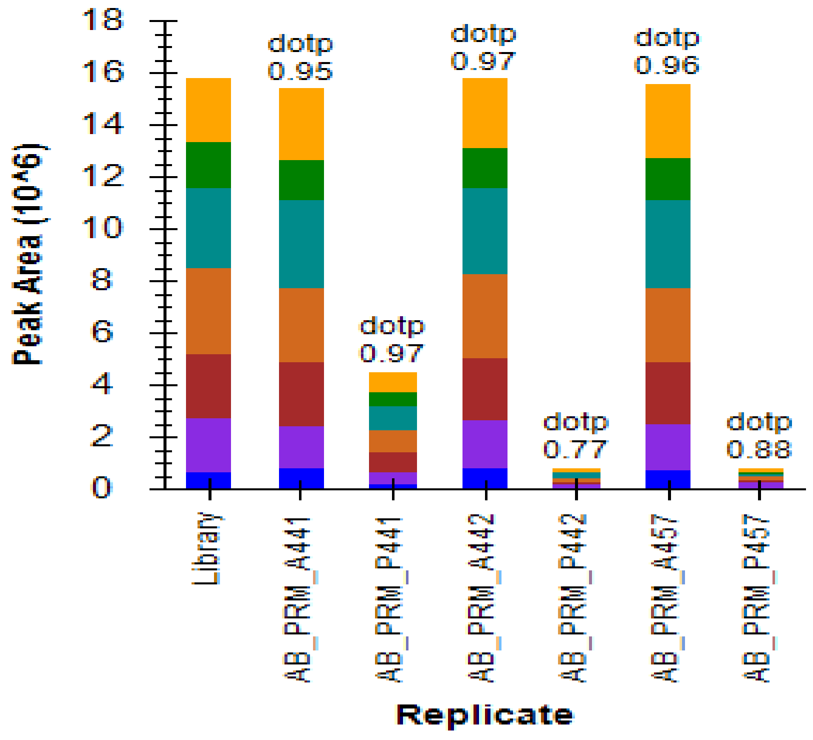

sp|P56937|DHB7_HUMAN

K.YATDLLSVALNR.N [198, 209]

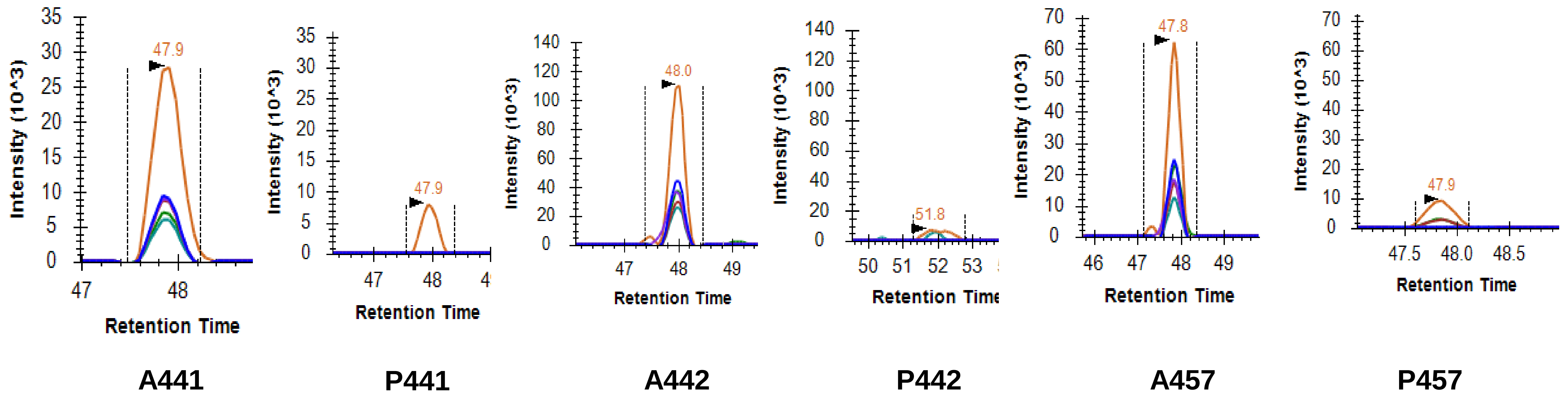

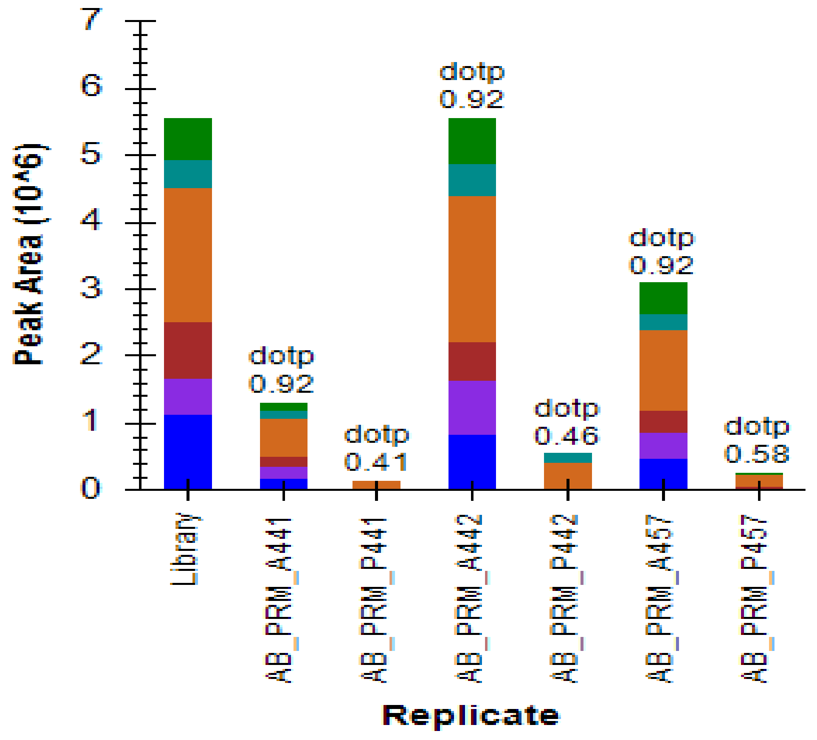

Supplement: Figure S7 [file mmc7.pdf]
