# Supplementary material for: The First Pituitary Proteome Landscape From Matched Anterior and Posterior Lobes for a Better Understanding of the Pituitary Gland
Source: Mol Cell Proteomics. 2022 Dec 5;22(1):100478. doi: 10.1016/j.mcpro.2022.100478 (PMC9877467; doi:10.1016/j.mcpro.2022.100478)
Supplement: Supplementary data [file mmc8.docx]

**The first Pituitary Proteome Landscape from matched anterior and posterior lobes for a better understanding of the Pituitary Gland**

**Running title**- First Pituitary Proteome draft of matched Anterior and Posterior lobes

Arghya Banerjee^1^, Deepatarup Biswas^1^, Abhilash Barpanda^1^, Ankit Halder^1^, Shamira Sibal^2^, Rohit Kattimani^3^, Abhidha Shah^4^, Anita Mahadevan^5^, Atul Goel^4^ and Sanjeeva Srivastava^1*^

**^1^**Department of Biosciences and Bioengineering, Indian Institute of Technology Bombay, Powai, Mumbai 400076, India

**^2^**Lokmanya Tilak Municipal Medical College, Dr. Babasaheb Ambedkar Road, Sion (West), Mumbai – 400022, India

**^3^**Anthony Claret School, Jalahalli, Bengaluru,560013, India

**^4^** Department of Neurosurgery at King Edward Memorial Hospital and Seth G. S. Medical College, Dr E Borges Road, Acharya Donde Marg, Parel, Opposite Tata & Wadia Hospital, Mumbai 400012, India

**^5^**Human Brain Bank, National Institute of Mental Health and Neuro Sciences (NIMHANS), Hosur Road / Marigowda Road, Bangalore – 560029, India

**Corresponding authors (Lead contact)**

**Sanjeeva Srivastava***- Department of Biosciences and Bioengineering, Indian Institute of Technology Bombay, Powai, Mumbai 400076; India: Email- [sanjeeva@iitb.ac.in](mailto:sanjeeva@iitb.ac.in)

**Contents**

**Supplementary figures:**

**Figure S1**- Shows a brief representation of the **Origin**: The anterior and posterior Pituitary develop separately in the embryo**; Development:** Ventral diencephalon, which expresses the proteins shown above, makes direct contact with oral ectoderm and induces the formation of Rathke's pouch. Ultimately somatotropes, lactotropes and thyrotropes are formed. Corticotropes and gonadotrophs are differentiated in the most ventral part of the gland. The dorsal region of the Rathke's pouch becomes the intermediate lobe, containing melanotropes; **Function:** Hormones secreted in the anterior and the posterior Pituitary and a wide range of functions with localizations are mentioned in the illustration.

**Figure S2- Panel A-** Shows Log10 Normalised Abundance plot of each group and Pearson Correlation Plot of anterior and posterior lobes of matched pair, **Panel B -**Venn Diagram of proteins found and anterior and posterior pituitary lobes. **Panel C-** Venn Diagram of Peptide Groups found modifications: Phospho and Met-Loss+ Acetyl (Protein N-term), **Panel D-** Bar Chart of PSMs Missed Cleavages and **Panel E-** Bar Chart of Pathway Protein group source.

**Figure S3-** Shows the count of proteins for biological process, count of proteins for molecular function and count of proteins for cellular components.

**Figure S4- Panel A**- Shows the chord plot of number proteins identified mapped to each chromosome. **Panel B-** Individual chromosomal plot to their number of proteins mapped.

**Figure S5- Panel A-**Depicts the structure predicted in AlphaFold along with the confidence score and the error rate of aligned residue, **Panel B-**The image represents the PDB structure of METTL26 that is predicted from both iTASSER and AlphaFold, **Panel C-**The predicted molecular function, biological process and cellular component of THEM6 and FSD1L from COFACTOR plotted against their CScoreGO.GO:0003824 depicts catalytic activ-ity, GO:0009987 represents the cellular process, GO:0071704 represents organic substance metabolic process, GO:0044237 rep-resents the cellular metabolic process, GO:1901575 depicts organic substance catabolic process, GO:0044238 stands for the cellular catabolic process. Similarly, the biological processes specific for FSD1L are GO:0065007, which depicts biological regulation, GO:0050789 represents regulation of biological process and for cellular component, GO:0044464 represents cell part, GO:0044424 stands for the intracellular part, GO:004326 is GO term for orga-nelle and GO:004444 is for the cytoplasmic part, **Panel D-** The scatter plot represents the count of uPE1 proteins mapped to a particular chromosome, **Panel E**- The bar chart represents the molecular function of METTL26 predicted by COFACTOR according to the CScoreGO.

**Figure S6-** String interaction analysis of differentially expressed protein and clustering in the anterior and posterior lobe.

**Figure S7-** Shows verification of Pituitary proteins H4, SOMA, THRAP3, TSHβ, AVP, OXT, POMC, GAS6, MIA2, PIT1, NPTX2, PCOLCE2, HTSF1 and DHB7.

**Supplementary Tables:**

All supplement data tables are available in Mendeley data under doi: 10.17632/4g8k9hvjcs.1

**Tables S1-** Instrument parameters of orbitrap fusion for Label-free quantification.

**Tables S2-** Instrument parameters of orbitrap fusion for parallel reaction monitoring.

**Tables S3-** Anterior vs Posterior Proteome discoverer output file for proteins.

**Table S4**- Anterior vs Posterior Proteome discoverer output file for peptide groups.

**Table S5-**Anterior vs Posterior Proteome discoverer output file for PSM.

**Table S6-** Proteome discoverer generated count of proteins in biological processes.

**Table S7-** Proteome discoverer generated count of proteins in molecular function.

**Table S8-** Proteome discoverer generated count of proteins in cellular components.

**Table S9-** Paired statistical analysis, excel sheet consisting of Proteins abundances volcano significant proteins from Metaboanalyst.

**Table S10-** Excel sheet containing pathway analysis of differentially significant proteins from Reactome, pathway analysis of protein upregulated in anterior and posterior individually.

**Table S11-** DEPs take for function clustering, Anterior enriched pathways with enrichment score and Posterior enriched pathways with enrichment score.

**Table S12-** Distribution data of 44 uPE1 protein and Coregulation study of METTL26 using ProteomeHD.

**Table S13-** String Interaction analysis of DEPs from Anterior and Posterior lobe.

**Table S14-** Excel sheet containing 33 proteins for heat map and box plot.

**Table S15-** Transitions monitored for each peptide quantified in the PRM quantification.
