# Supplementary material for: The First Pituitary Proteome Landscape From Matched Anterior and Posterior Lobes for a Better Understanding of the Pituitary Gland
Source: Mol Cell Proteomics. 2022 Dec 5;22(1):100478. doi: 10.1016/j.mcpro.2022.100478 (PMC9877467; doi:10.1016/j.mcpro.2022.100478)
Supplement: Table S1 [file mmc9.pdf]

Supplementary Table 1- LFQ method

Method Summary

Method Settings

Application Mode: **Peptide**
Method Duration (min): **240**

Global Parameters

Ion Source

Ion Source Type: **NSI**
Spray Voltage: **Static**
Positive Ion (V): **1900**
Negative Ion (V): **0**

Positive Ion

Positive Ion	
Time (min)	Voltage (V)

Negative Ion

Negative Ion	
Time (min)	Voltage (V)

Sweep Gas (Arb): **0**
Ion Transfer Tube Temp (°C): **275**
Use Ion Source Settings from Tune: **False**
FAIMS Mode: **Not Installed**

MS Global Settings

Default Charge State: **1**
Internal Mass Calibration: **User-defined Lock Mass**
Current Lock Mass: **Current**

Positive Ion

Positive Ion
m/z
445.12003

Negative Ion

Negative Ion

m/z

Experiment#1 [MS]

Start Time (min): **0**
End Time (min): **240**
Cycle Time (sec): **3**

Master Scan:

MS OT

Detector Type: **Orbitrap**
Orbitrap Resolution: **60000**
Mass Range: **Normal**
Use Quadrupole Isolation: **True**
Scan Range (m/z): **375-1700**
RF Lens (%): **60**
AGC Target: **4.0e5**
Maximum Injection Time (ms): **50**
Microscans: **1**
Data Type: **Profile**
Polarity: **Positive**
Source Fragmentation: **Disabled**
Scan Description:

Filters:

MIPS

Monoisotopic Peak Determination: **Peptide**

Charge State

Include charge state(s): **2-6**
Include undetermined charge states: **False**
Include charge states 25 and higher: **False**

Dynamic Exclusion

Exclude after n times: **1**
Exclusion duration (s): **40**
Mass Tolerance: **ppm**
Low: **10**
High: **10**
Exclude Isotopes: **True**
Perform dependent scan on single charge state per precursor only: **False**

Intensity

Filter Type: **Intensity Threshold**
Intensity Threshold: **5.0e3**

Data Dependent

Data Dependent Mode: **Cycle Time**
Time between Master Scans (sec): **3**

Scan Event Type 1:

Scan:

ddMS^2 OT HCD

Isolation Mode: **Quadrupole**
Isolation Window (m/z): **1.2**
Isolation Offset: **Off**
Activation Type: **HCD**
Collision Energy Mode: **Fixed**
HCD Collision Energy (%): **30**
Detector Type: **Orbitrap**
Scan Range Mode: **Auto: m/z Normal**
Orbitrap Resolution: **15000**
First Mass (m/z): **100**
AGC Target: **1.0e4**
Inject Ions for All Available Parallelizable Time: **True**
Maximum Injection Time (ms): **30**
Microscans: **1**
Data Type: **Centroid**
Use EASY-IC™: **False**
Scan Description:
