# Supplementary material for: The First Pituitary Proteome Landscape From Matched Anterior and Posterior Lobes for a Better Understanding of the Pituitary Gland
Source: Mol Cell Proteomics. 2022 Dec 5;22(1):100478. doi: 10.1016/j.mcpro.2022.100478 (PMC9877467; doi:10.1016/j.mcpro.2022.100478)
Supplement: Table S2 [file mmc10.pdf]

Method Summary

Method Settings

Application Mode: **Peptide**
Method Duration (min): **60**

Global Parameters

Ion Source

Use Ion Source Settings from Tune: **True**
FAIMS Mode: **Not Installed**

MS Global Settings

Default Charge State: **3**
Internal Mass Calibration: **Off**

Experiment#1 [MS]

Start Time (min): **0**
End Time (min): **60**

Master Scan:

MS OT

Detector Type: **Orbitrap**
Orbitrap Resolution: **60000**
Mass Range: **Normal**
Use Quadrupole Isolation: **True**
Scan Range (m/z): **350-1700**
RF Lens (%): **60**
AGC Target: **4.0e5**
Maximum Injection Time (ms): **50**
Microscans: **1**
Data Type: **Profile**
Polarity: **Positive**
Source Fragmentation: **Disabled**
Scan Description:

Experiment#2 [tMSn]

Start Time (min): **0**
End Time (min): **60**

Master Scan:

tMS^2 OT HCD

MS^n Level (n): **2**
Multiplex Ions: **False**
Isolation Mode: **Quadrupole**
Isolation Window (m/z): **1.6**
Activation Type: **HCD**
HCD Collision Energy (%): **30**
Stepped Collision Energy: **True**
+/- HCD Collision Energy (%): **5**
Detector Type: **Orbitrap**
Orbitrap Resolution: **30000**
Mass Range: **Normal**
Scan Range (m/z): **350-2000**
RF Lens (%): **60**
AGC Target: **5.0e4**
Inject Ions for All Available Parallelizable Time: **True**
Maximum Injection Time (ms): **40**
Microscans: **1**
Data Type: **Centroid**
Polarity: **Positive**
Source Fragmentation: **Disabled**
Use EASY-IC™: **False**
Loop Control: **Time**
Time (sec): **3**
Scan Description:
Include Start/End Times: **False**

Mass List Table

Mass List Table				
Compound	Formula	Adduct	m/z	z
			1073.0448	2
			655.3486	2
			659.3435	2
			579.3249	2
			551.7999	2
			807.4541	2
			917.4635	2
			706.3988	2
			765.9009	2
			882.4048	2
			601.307	2
			658.8257	2
			578.8322	2
			818.4407	2
			727.384	2
			663.8324	2

			577.8608	2
			505.7725	2
			423.7138	2
			895.9496	2
			884.4436	2
			702.7973	2
			590.8141	2
			495.2926	2
			655.8549	2
			1171.5706	2
			1028.1037	2
			745.3495	2
			577.3162	2
			993.4527	2
			693.3126	2
			492.7955	2
			570.762	2
			548.2275	2
			747.8334	2
			977.0531	2
			801.9191	2
			688.3192	2
			863.364	2
			660.8054	2
			678.7816	2
			642.3877	2
			805.9265	2
			467.7368	2
			537.247	2
			1210.9553	2
			798.9388	2
			428.7298	2
			962.9118	2
			1034.4498	2
			528.732	2
			1056.9917	2

			510.2288	2
			567.3319	2
			546.2429	2
			504.2708	2
			526.2984	2
			701.8497	2
			544.2984	2
			553.3037	2
			700.3932	2
			470.7824	2
			868.4669	2
			533.7529	2
			802.3815	2
			755.935	2
			656.81	2
			813.3919	2
			468.7247	2
			746.3735	2
			572.3095	2
			819.388	2
			491.248	2
			553.2673	2
			874.4341	2
			506.3135	2
			692.815	2
			520.8268	2
			718.417	2
			550.8191	2
			556.8191	2
			589.788	2
			487.8053	2
			489.779	2
			690.3311	2
			821.9033	2
			563.3006	2
			538.7683	2

			662.859	2
			500.7747	2
			452.7133	2
			618.8202	2
			623.3435	2
			637.3612	2
			706.793	2
			603.2664	2
			691.3245	2
			894.913	2
			774.3616	2
			564.804	2
			811.4038	2
			668.3382	2
			497.2298	2
			543.2414	2
			501.7587	2
			737.8573	2
			892.3663	2
			498.7171	2
			761.3941	2
			727.3753	2
			596.3346	2
			734.8877	2
			733.8145	2
			842.8867	2
			751.8961	2
			527.246	2
			747.8704	2
			656.8359	2
			707.8699	2
			693.3672	2
			806.8966	2
			474.7611	2
			587.2771	2
			668.367	2

			500.2771	2
			503.2718	2
			1024.4913	2
			472.7693	2
			723.8466	2
			634.298	2
			576.8116	2
			640.8823	2
			593.3148	2
			986.5197	2
			548.8273	2
			711.3689	2
			482.7404	2
			723.3401	2
			502.2332	2
			636.3005	2
			847.9048	2
			635.8613	2
			426.7525	2
			523.7795	2
			627.8004	2
			518.2699	2
			662.3726	2
			482.7585	2
			778.3949	2
			492.2288	2
			544.7904	2
			743.893	2
			453.2638	2
